# On the Value of In Vitro Cell Systems for Mechanobiology from the Perspective of Yes-Associated Protein/Transcriptional Co-Activator with a PDZ-Binding Motif and Focal Adhesion Kinase and Their Involvement in Wound Healing, Cancer, Aging, and Senescence

**DOI:** 10.3390/ijms241612677

**Published:** 2023-08-11

**Authors:** Thorsten Steinberg, Martin Philipp Dieterle, Imke Ramminger, Charlotte Klein, Julie Brossette, Ayman Husari, Pascal Tomakidi

**Affiliations:** 1 Center for Dental Medicine, Division of Oral Biotechnology, Medical Center—University of Freiburg, Faculty of Medicine, University of Freiburg, Hugstetterstr. 55, 79106 Freiburg, Germany; 2 Faculty of Biology, University of Freiburg, Schaenzlestr. 1, 79104 Freiburg, Germany; 3 Center for Dental Medicine, Department of Orthodontics, Medical Center—University of Freiburg, Faculty of Medicine, University of Freiburg, Hugstetterstr. 55, 79106 Freiburg, Germany

**Keywords:** mechanotransduction, mechanobiology, mechanosignaling, focal adhesion kinase, yes-associated protein, tissue homeostasis, in vitro cell system(s), aging, senescence, cancer, wound healing

## Abstract

Mechanobiology comprises how cells perceive different mechanical stimuli and integrate them into a process called mechanotransduction; therefore, the related mechanosignaling cascades are generally important for biomedical research. The ongoing discovery of key molecules and the subsequent elucidation of their roles in mechanobiology are fundamental to understanding cell responses and tissue conditions, such as homeostasis, aging, senescence, wound healing, and cancer. Regarding the available literature on these topics, it becomes abundantly clear that in vitro cell systems from different species and tissues have been and are extremely valuable tools for enabling the discovery and functional elucidation of key mechanobiological players. Therefore, this review aims to discuss the significant contributions of in vitro cell systems to the identification and characterization of three such key players using the selected examples of yes-associated protein (YAP), its paralog transcriptional co-activator with a PDZ-binding motif (TAZ), and focal adhesion kinase (FAK) and their involvement in wound healing, cancer, aging, and senescence. In addition, the reader is given suggestions as to which future prospects emerge from the in vitro studies discussed herein and which research questions still remain open.

## 1. Introduction

Mechanobiology encompasses all of the molecular processes of the cells in our body tissues that are involved in sensing mechanical signals at the cell and nuclear membranes, intracellularly processing them, and then converting them into cellular behavioral responses through the regulation of the genome and proteome. Ultimately, all of these processes of perception, processing, and regulation result in a cellular response to the environmental mechanical stimulus. This, in turn, is expressed as the modulation of cell functions that we can detect and are further modulated by cell behavior. To provide just a few examples, cellular behavior is determined by essential cellular functions, such as proliferation and differentiation, which, in turn, represent cornerstones of tissue homeostasis in the human body [1,2].

To establish tissue homeostasis and maintain it throughout life, cells from solid body tissues, which include all tissues except blood and lymph, synthesize a so-called extracellular matrix (ECM). The ECM, as a natural cell environment, shows distinct stiffness and a spatial arrangement of contact points for cell adhesion that depends on its molecular composition and the resulting three-dimensional architecture. A fundamental study in the field of cell differentiation in response to matrix elasticity/rigidity is the work of Engler et al. [3], who were able to show, for the first time, the influence of different microenvironments on the lineage specificity of MSCs. This, thus, inspired a whole generation of researchers in this field, e.g., Dupont et al., who discovered that YAP/TAZ are the nuclear relays of mechanical signals exerted by ECM rigidity [4]. This rigidity of the ECM and the spatial patterning of cell adhesion points represent two essential biomechanically and, thus, mechanobiologically relevant influencing variables on cells, which later determine the cell response. In addition, viscoelasticity has been found to be a universal characteristic of living tissues and ECMs. In response to a mechanical perturbation, viscoelastic materials exhibit an instantaneous elastic response, which is characteristic of purely elastic solids, followed by a time-dependent mechanical response and energy dissipation or loss. Viscoelastic materials “creep”, or deform in a time-dependent manner, in response to the application of an external stress stimulus or load and undergo stress relaxation or reduce stress levels in a time-dependent manner in response to a step deformation [5]. To more precisely examine the connections among the aforementioned variables under defined conditions, model surroundings or model surfaces made of elastic substrates, such as polydimethylsiloxane (PDMS), are helpful since they can be modularly calibrated with regard to both elasticity and the distances between cell adhesion points [6]. Regarding the mimetics of tissue matrices, hydrogels with variations in their viscous properties have been employed, which proved that, in the context of chondrogenesis, the viscous nature of the matrix can be harnessed to direct cell fate [7]. Whatever the nature of these biomechanical factors is, they are recognized by cells with the help of adhesion molecules, such as integrins, so the cells can attach to the ECM and form contact structures, termed focal complexes or focal adhesions (FAs).

Moreover, biomechanical signals can also be transmitted when cells establish contact with neighboring cells, so mechanotransduction integrates horizontal contact structures, such as cadherin-based adherens junctions (AJs), in addition to vertically acting FAs. Both FAs and AJs contain highly ordered ultrastructural collections of molecules like integrins (e.g., integrin β1) and cadherins (e.g., E-cadherin in epithelia) [1,8], which, as mechanosensors and mechanotransducers, ensure that the biomechanical force emanating from the ECM or adjacent cells is perceived at the plasma membrane. Following perception, biomechanical cues are converted into biochemical signals and transported through the cytoplasm into the cell nucleus. This happens with the help of specific signaling molecules, which then modulate the regulation of genes that are important for the cellular response. Hence, all molecule-driven processes that contribute to the conversion of a mechanical signal into a cellular response are subsumed under the term mechanotransduction [2].

Fundamentally, mechanobiology is reciprocal in nature. In addition to mechanotransduction from the ECM into the cell interior, the so-called outside-in signaling, the cell also exerts biomechanical forces on its environment and, thus, regulates the biomechanical properties of the matrix. This force is generated and exerted by the cells themselves and is used to interpret biophysical cues such as rigidity/(visco)elasticity and the spatial organization of adhesion sites (ligand spacing and nanotopography) by force loading in molecular clutches, as shown by recent articles [9,10,11]. It regulates not only the biomechanical properties of the cells but also the cellular responses to the biophysical cues controlling cell behavior and fate.

For the state of tissue homeostasis, this means that outside-in and inside-out signaling requires a finely tuned balance, since imbalances in this bi-directionality result in disturbances of the physiology of cells and tissues [12]. This is important since many studies focus on cells cultured in 2D on top of elastic substrates with a range of stiffnesses. However, cells often interact with ECMs in vivo in a 3D context, and cell–ECM interactions and the mechanisms of mechanotransduction in 3D can differ from those in 2D. Furthermore, cell–matrix interactions are dynamic owing to matrix remodeling. Hence, ECM stiffness, viscoelasticity, and degradability often play a critical role in regulating cell behavior in 3D. The mechanisms of 3D mechanotransduction include traditional integrin-mediated pathways that sense mechanical properties and more recently described mechanosensitive ion-channel-mediated pathways that sense 3D confinement, with both converging on the nucleus for the downstream control of transcription and the phenotype. All of these aspects would have been excluded from consideration in 2D environments and draw a clear line between the two approaches. This depicts the limitations of 2D in vitro studies [13], although it has been shown that nanotopographical features can decisively influence cell mechanotransduction and behavior [14]. Since the present review does not specifically deal with the topics described above, we would like to refer interested readers to the review articles written by Miller et al. [15] and Saraswathibhatla et al. [13], who examine these topics extensively.

Due to the complexity of the in vivo situation, which includes interactions between cells from different tissues, this review focuses on in vitro cell systems, which are discussed in detail in the following chapters. This is because they greatly simplify the complexity of the in vivo situation and thereby enable direct causal relationships between mechanobiologically relevant extracellular stimuli and their effects on gene and protein regulation, as well as cell functions. The importance of simplification was demonstrated, for example, in a previous study on the mechanobiologically governed wound healing of epithelia (see also YAP/TAZ and FAK in wound healing, Section 2.3). In this study, using in vitro keratinocyte monolayer cultures, Di Russo et al. showed that the nanospacing of integrin α5β1 on the ECM side, regardless of its rigidity, is crucial for the migration of keratinocytes, which, on the other hand, is essential for wound healing [16]. In this context, it is most noteworthy that the FAs themselves and many of the molecular components of focal contacts, such as vinculin and talin, have been discovered in cells isolated from donor tissues and further explored chronologically with regard to their biological functions and molecular characteristics [17,18].

With regard to their role as focal contact components, it has been questioned as to whether they are involved in production or whether they simply provide chemical signals by possessing phosphorylation capacity, with binding sites for phosphate residues, or change in conformation [19]. According to the previous understanding of their definition in mechanobiology, these properties determine whether the individual components of focal contacts act as mechanosensors (e.g., integrins, talin, and vinculin), mechanotransducers (e.g., FAK), or actin cytoskeleton regulators (e.g., vasodilator-stimulated phosphoprotein (VASP) and zyxin). FAK’s signaling cascade starts with the autophosphorylation of tyrosine residue 397 upon a conformational change. Therefore, FAK can be considered both a mechanosensor and a mechanotransducer, as can be shown for keratinocytes, for example, in the context of fibrogenic gene expression [20], whereby the mode of FAK activation represents the trigger of mechanotransduction. At this point, it should be noted that certain molecules are classified as mechanosensors or mechanotransducers based on the characteristics just described. However, this minimalist view is currently in a state of upheaval. This upheaval is based on the increasing complexity of the molecules involved in mechanobiology. To be able to make a clear distinction between the functions of mechanobiologically relevant molecules, it is recommended, in this context, to make a clearer delimitation between (i) molecules that sense and record the mechanical signal, (ii) those that transport it to the cell nucleus, and (iii) those that activate mechanosensitive target genes. This aspect, which will be very important for mechanobiology in the future, has been taken up and discussed in a contemporary review [21].

Within cell-to-cell contact-forming AJs, the catenins, including β-catenin (alternatively γ-catenin in some cell types) and the actin-binding protein α-catenin, together with the cadherin–catenin complex stabilizer p120, are characterized as mechanosensors (α-catenin [22]) and mechanotransducers (β-catenin [23]). In addition, so-called shuttling mechanotransducers have been discovered, as exemplified by the transcription co-activator YAP (Yes-associated protein) and its homolog transcriptional co-activator with a PDZ-binding motif (TAZ). The name arises from the fact that it can switch back and forth between the cytoplasm, where it is transcriptionally inactive, and the cell nucleus, where it is transcriptionally active [21,24].

Furthermore, contact structures between cells that are essential for mechanobiology, in addition to AJs, are desmosomes and tight junctions. Although not the subject of this review, they are mentioned for the sake of completeness. In contrast to other cell-to-cell contacts, desmosomes are intracellularly linked to IFs (intermediate filaments), while tight junctions are coupled to actin and microtubules via ZO proteins (zonula occludens). Since the interplay of all these contact structures in conjunction with the mechanosensory or mechanotransductive molecules decides which response the cell ultimately produces to the biomechanical environmental stimulus, it is obvious that disruptions in the coordination of the molecules involved have consequences for cell physiology and, in a broader sense, for tissue homeostasis.

It is important, for a holistic view of mechanobiology, including mechanotransduction within both the cytoplasm and the nucleus, that all relevant contact structures and ion channels are intracellularly connected to the cytoskeleton. The cytoskeletal binding partners, i.e., actin, tubulin, and IFs, in turn, connect to certain proteins on and in the nuclear membrane, which are linked to nuclear-associated IFs, the lamins, which, in turn, are connected to the chromatin [25]. Overall, via these connections, mechano-biomechanical signals, after their passage through the cytoplasm, lead to the modulation of the activity of intranuclear mechanoresponsive genes, and this gene modulation ultimately determines the cell response.

In view of the far-reaching importance of mechanobiology for the cells and tissues of our bodies, this review discusses distinct key molecular players in physiological and non-physiological situations, such as wound healing or cancer. The focus will be on the valuable contributions of in vitro experiments and in vitro cell systems to the discovery of master molecules and the elucidation of their biological functions. The knowledge gained from such in vitro studies is the basis for learning to distinguish healthy from diseased cell and tissue situations from the perspective of mechanobiology. This, in turn, is essential to increasingly identify biomarkers for diagnostics or develop them for the alleviation of diseases and concepts for future therapy options. Therefore, we would like to take the reader on the exciting journey of how cell research has used in vitro cell systems in recent years to uncover and characterize molecular networks in which three of the mechanobiological key players, namely, YAP, TAZ, and FAK, are embedded.

All of these examples illustrate the invaluable importance of cell-culture-based in vitro test systems for biomedical research in general and especially in the context of unraveling the molecular basis of not only tissue-immanent processes, such as aging, wound healing, and cancer, but also disease prevention (see Supplementary Materials). For this reason, it is the central concern of this review to take a closer look at the application of such test systems by focusing on some of the key players in mechanobiology. Hence, regardless of whether it was the primary literature or review articles, the literature search was based on a systematic approach, which involved the use of specific search terms for the respective overall or partial aspects. This procedure was used in all sections, with a focus on (i) history, i.e., identification and characterization of YAP/TAZ and FAK, (ii) aging and senescence, (iii) wound healing, (iv) cancer, and (v) diagnosis and therapy.

## 2. Cell-Culture-Based In Vitro Test Systems for Biomedical Research

### 2.1. In Vitro Systems That Helped to Identify and Characterize YAP/TAZ and FAK as Mechanotransducers

#### 2.1.1. YAP/TAZ

**Figure 1:**
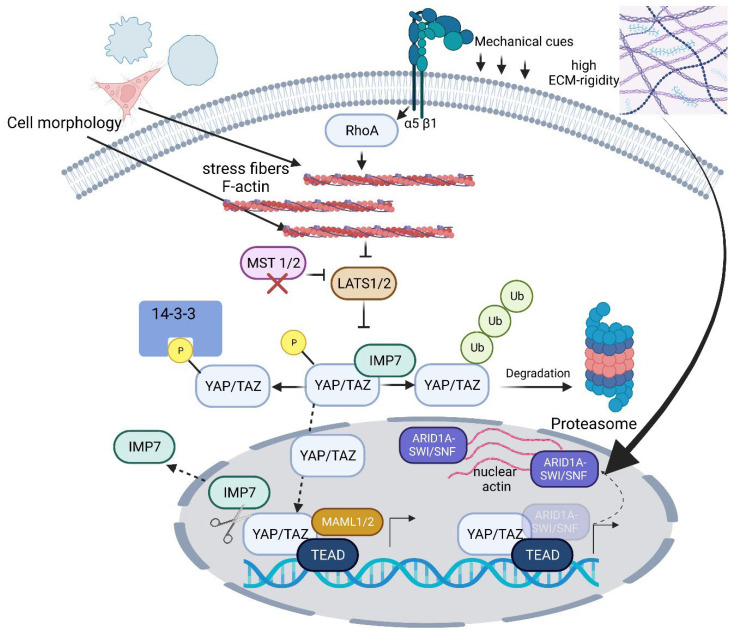
Schematic overview of YAP/TAZ signaling in mechanotransduction. YAP/TAZ illustrated as nuclear relays of mechanical signals exerted by the ECM rigidity and cell shape, which requires Rho GTPase (RhoA) activity and tension of the actomyosin cytoskeleton. YAP/TAZ molecules are inhibited through phosphorylation by the Hippo-innate large tumor suppressor kinase 1/2 (LATS1/2). In the case of YAP, this occurs through the LATS1/2-dependent phosphorylation of YAP at serine residue 127. Following this phosphorylation, YAP interacts with the 14-3-3 protein and is thus retained in the cytoplasm and subsequently ubiquitinated and degraded. For the co-transcription function, YAP shuttles back and forth between the nucleus and the cytoplasm. Therefore, it must enter the nucleus after being released from the cytoplasmic 14-3-3 protein. Its entry is mediated by nuclear transport receptors (NTRs), and YAP specifically binds to Importin 7 (IMP7) as an NTR. YAP/IMP7 interaction requires the inactivation of MST1/2, which, in turn, activates the YAP-inhibiting LATS kinase. The permanent function of YAP as a co-transcriptional activator requires its interaction with MAML1/2, acting as transcriptional co-activators by forming a trimeric complex with YAP/TAZ and TEAD to induce the gene transcription of YAP/TAZ-specific genes. The YAP/TAZ complex formation dynamics are regulated by a protein complex called ARID1A-SWI/SNSF. This complex binds to YAP/TAZ, preventing interaction with TEAD. Moreover, in response to high ECM rigidity, nuclear actin increasingly polymerizes and binds to the ARID1A-SWI/SNF complex. This interaction between the complex and nuclear actin facilitates the progressive release of YAP/TAZ from the AR-ID1A-SWI/SNF complex and, therefore, allows for YAP/TAZ interaction with TEAD to initiate the transcription of target genes. The schematic was created with BioRender.com.

Yes-associated protein, YAP, was originally identified, characterized, and cloned as a proline-rich phosphoprotein that binds to the SH3 domain of the Yes proto-oncogene product by employing an in vitro cell system comprising chick embryonic fibroblasts (CEFs) [26]. The same holds true for the Yes homolog TAZ, which was identified as a transcriptional co-activator regulated by interactions with 14-3-3 and PDZ domain proteins in HeLa (Henrietta Lacks—a cervix-carcinoma-derived cell line) cells [27]. In 2011, Dupont et al. [4] published an article that discusses YAP/TAZ as nuclear relays of mechanical signals exerted by the ECM rigidity and cell shape, which requires Rho GTPase activity and the tension of the actomyosin cytoskeleton. In doing so, they identified YAP/TAZ as sensors and mediators of mechanical cues—important for mesenchymal stem cell differentiation and endothelial cell (EC) survival [4]. In this context, shear forces also play an important role in the activation of YAP/TAZ, as Wang et al. [28] found that disturbed flow without a clear direction, but not laminar flow with a clear direction, activates YAP/TAZ to promote the proliferation and inflammation of vascular endothelial cells. For macrophages, it has been shown that they adhere to the extracellular matrix within tissues and that the adhesive microenvironment tunes the macrophage inflammatory response through the transcriptional co-activator YAP. Hence, researchers discovered that the adhesion of macrophages to soft hydrogels reduces inflammation when compared to adhesion to stiff materials and is associated with reduced YAP expression and nuclear localization. Furthermore, the depletion of YAP inhibits macrophage inflammation, whereas the overexpression of active YAP increases inflammation [29]. However, recent research has highlighted biphasic relationships between cell behavior and substrate stiffness. Here, YAP mechanosensing shows a biphasic response depending on both substrate stiffness and RGD ligand spacing, and, additionally, Oria et al. [11] revealed that the spatial sensing between integrin-containing adhesion complexes and the nanometer-scale distribution of ECM ligands and downstream YAP regulation is even more complex. Since YAP is a crucial mechanosensitive transcriptional co-activator involved in regulating cell behaviors, such as differentiation, this could be associated with how high-strength gels influence chondrogenesis [30]. In 2011 again, Wada et al. [31] showed that, in in vitro cell cultures based on immortalized mouse fibroblasts (NIH3T3 cells) and neoplastic mouse epithelial mammary gland cells (MTD-1A), the nuclear-bound activity of YAP depends on the cell morphology and the proportion of stress-fiber-containing F-actin. The cell morphology was modified by varying the extracellular environment by creating square micro-domains of different sizes (edge length: 20, 50, or 70 µm). A high proportion of stress fibers at a low cell density and flat, spread-out cells suppressed the Hippo signaling pathway described below, leading to the phosphorylation of YAP, which then inhibited its nuclear translocation. The important role of stress fibers in the activation of YAP could be demonstrated, among other things, by using stress-fiber disruptors such as cytochalasin D. Conversely, the presence of stress fibers was also reduced in round cells, and YAP was found to be increased in the inactive form in the cytoplasm [31]. The results show that the activity of YAP is regulated via morphology and, downstream, by actin stress fibers and, thus, the inherent biomechanics of the cell. The extracellular environment (Figure 1), in turn, controls the cell biomechanics.

YAP and TAZ are transcription co-activators since they do not have their own DNA-binding motifs. Their regulation, i.e., activation or inactivation, takes place via phosphorylation and dephosphorylation. They are effector molecules of the canonical Hippo signaling pathway. The Hippo signaling pathway is essential for tissue homeostasis and other processes, such as development and tissue regeneration. Both molecules are inhibited through phosphorylation by the Hippo-innate large tumor suppressor kinase 1/2 (LATS1/2). In the case of YAP, this occurs through the LATS1/2-dependent phosphorylation of YAP at serine residue 127. Following this phosphorylation, YAP interacts with the 14-3-3 protein and is thus retained in the cytoplasm and subsequently ubiquitinated and degraded (Figure 1). This pathway was initially discovered in Drosophila melanogaster as a key regulator of tissue growth [32]. The core of the Hippo pathway in mammals consists of a kinase cascade, including MST1/2 and LATS1/2, as well as downstream effectors, particularly the transcriptional co-activators YAP and TAZ. These core components of the Hippo pathway control transcriptional programs involved in cell proliferation, survival, mobility, stemness, and differentiation [32]. Further knowledge on the regulation of TAZ and YAP was resolved in so-called knockout cell lines based on human embryonic kidney (HEK293T) cells, in which the genes for numerous Hippo-signaling-pathway-associated molecules, including TAZ, were deactivated by the CRISPR/Cas9 system [33]. In a follow-up study, Plouffe et al. [34], using the same in vitro cell system and technique of CRISPR/Cas9 gene knockout (KO) of YAP, TAZ, and YAP/TAZ, showed that YAP has a stronger effect on cell physiology compared to TAZ. The cell physiological parameters included cell functions such as cell spread, cell volume, glucose uptake, and granule content, as well as proliferation and migration. From this, the authors concluded that TAZ and YAP are master regulators of multiple physiologically relevant cell functions but that YAP dominates TAZ here due to its stronger influence on the mentioned functions [34]. To date, however, it has not been clarified on what the dominance of YAP over TAZ in relation to the cell functions just described is based. Today, structural intramolecular differences between YAP and TAZ are discussed. These relate, among other things, to the TEAD-binding domain. This domain appears to be more hydrophobic in character in YAP. In addition, TAZ can form a hetero-tetramer complex with TEAD [35], which can affect DNA target selectivity and thereby induce greater expression of certain target genes [34,36].

For the co-transcription activator YAP, which shuttles back and forth between the nucleus and the cytoplasm, to be able to perform its function at all, it must enter the nucleus after being released from the cytoplasmic 14-3-3 protein. The mechanism behind this process has so far been largely unclear, but recent research shows that so-called NTRs (nuclear transport receptors) are apparently involved, which can pass through nuclear pore complexes in an energy-dependent manner. Due to their sieving function, these pore complexes are difficult to pass through by molecules larger than 30 kDa, except NTRs. For an NTR to function, it must interact with a Ran-related or Ras-like nuclear protein (Ran), capable of binding GTP (guanosine triphosphate). For this purpose, each NTR has a RanGTP-binding motif, and the gradient between RanGTP (nucleus) and Ran guanosine diphosphate (GDP, cytoplasm) determines the direction of transport at the nuclear envelope, i.e., import or export. Using different cell lines, such as human retinal pigment epithelial-1 (RPE-1) cells, primary mouse aortic smooth muscle cells (MOVAS), and HEK293 (parental HEK cell line), as well as primary mesenchymal stem cells (MSCs), Garcia-Garcia et al. [37] showed that YAP specifically binds to Importin 7 (IMP7) as an NTR to enter the nucleus. An aspect that is important to know is that YAP regulates the mechanoresponsiveness of IMP7 by forming a complex with IMP7 (Figure 1). Furthermore, the same group showed that YAP, as an IMP7 binding partner, is dominant over other IMP7 binding partners, which include, for example, Smad3 (Small worm phenotype in C. Elegans and Mother against Decapentaplegic (MAD) in Drosophila 3) and MAPK1 (mitogen-activated protein kinase 2) [38]. YAP/IMP7 interaction apparently requires the inactivation of the Hippo-signaling-pathway-innate kinase MST1/2, which, in turn, activates the YAP-inhibiting LATS kinase [39]. Garcia-Garcia et al. were able to show these mechanistic relationships using cell-stretching experiments, cell substrates with different rigidities, and experiments with actomyosin-complex-perturbating agents. From their experiments, they concluded that extra- and intracellular biomechanical signals are indirectly involved in the regulation of the nuclear import process of YAP and that YAP dominates over other IMP7 binding partners [37]. Further knowledge on force-related YAP nuclear entry was discovered by Elosegui-Artola et al. [40], who found that force applied to the nucleus directly drives YAP nuclear translocation by decreasing the mechanical restriction of nuclear pores to molecular transport. In detail, exposure to a stiff environment leads cells to establish a mechanical connection between the nucleus and the cytoskeleton, allowing forces exerted through focal adhesions to reach the nucleus. Force transmission then leads to nuclear flattening, which stretches nuclear pores, reduces their mechanical resistance to molecular transport, and increases YAP nuclear import [40].

Once in the nucleus, the permanent biological function of YAP as a co-transcriptional activator apparently requires its interaction with molecules that promote its retention in the nucleus. Kim et al. [41] showed that Mastermind-line 1 and 2 (MAML1/2) displayed these properties. Both molecules were originally identified as pivotal co-activators of Notch-dependent transcription, whereby Notch acts as a membrane-bound transcription factor (TF) that is released in response to ligand binding by two proteases acting sequentially [42]. Within MAML1/2, Kim et al. discovered an evolutionarily conserved proline-rich PPxY interaction motif that physically binds to a protein module containing two conserved tryptophans (W, called the WW domain) in YAP/TAZ. This binding leads to the promotion of the nuclear retention of YAP/TAZ and downstream transcriptional activity. In this context, it is important to mention that MAML1/2 act as transcriptional co-activators by forming a trimeric complex with YAP/TAZ and TEAD to induce the gene transcription of YAP/TAZ-specific genes (Figure 1). Furthermore, it is worth noting that the mutation of a putative MAML nuclear localization signal (NLS) sequence altered YAP/TAZ activity to a suppressed state, indicating the pivotal role of MAML1/2 in regulating YAP/TAZ nuclear localization. Interestingly, the amount of MAML1/2, like that of YAP, correlated with the cell density. Both molecules were present in high abundance in the nucleus when the cell density was low. The aspect of cell density is of further importance for MAML1/2-dependent YAP regulation since MAML1 is regulated by a microRNA (miR), in this case, miR-30c. At high cell densities, there is a large amount of miR-30c, which, in turn, has a negative regulatory effect on the amount of MAML1 and thus affects the abundance of YAP in the cell nucleus. The key role of miR-30c in the regulation of MAML1 could be shown by inhibition experiments with a miR-30c-specific inhibitor. In fact, in miR-30c-treated cells that showed a reduction in MAML1, this reduction could be reversed under the influence of the inhibitor. Accordingly, the study shows that these findings have not only contributed to a better understanding of the regulation of YAP but have also aided in identifying the miR-30c-MAML-YAP axis as a possible therapeutic target for future anti-cancer strategies. To gain insights into the regulation of the nuclear abundance of YAP, Kim et al. [41] used numerous cell lines, including HeLa, Huh7, HT29, Caco2, DLD-1, SW480, and HCT116 cells, as in vitro models for their tests. This work demonstrates the complex regulatory mechanism behind YAP/TAZ nuclear translocation and transcriptional control, and it has been additionally stated that mechanical/biomechanical forces, i.e., stiff ECM, contribute significantly to YAP availability in the nucleus [4], allowing for its interaction with MAML molecules and TEAD and promoting transcription [41], raising more questions on the regulation of these interactions. Therefore, regarding the interaction of YAP with TEAD after nuclear translocation, the question arises as to how this interaction is regulated. By using in vivo approaches in conjunction with various cell lines, for instance, HEK293T cells, in vitro experiments revealed that a possible explanation for this is provided by a protein complex called ARID1A (AT-rich interactive domain-containing protein 1A)–SWI/SNSF (SWItch/Sucrose Non-Fermentable). This complex binds to YAP/TAZ, preventing their interaction with TEAD (Figure 1). On the other hand, if the extracellular environment shows high stiffness, as, for example, represented by high ECM rigidity, nuclear actin increasingly polymerizes and binds to the ARID1A–SWI/SNF complex. This interaction between the complex and nuclear actin facilitates the progressive release of YAP/TAZ from the ARID1A–SWI/SNF complex and, therefore, allows for YAP/TAZ interaction with TEAD to initiate the transcription of target genes [43] (Figure 1).

In 2018, Kofler et al. [44] used epithelial kidney cells (LLC-PK1) obtained from a male pig and were also able to identify and characterize a diffusion-independent and thus directed mechanism of the back and forth oscillation of TAZ between the cytoplasm and nucleus. By running transfection experiments, which combine fusion plasmids, e.g., TAZ and plasmids with directed mutations in molecules, such as LATS, and targeted deletions, for example, in certain regions of TAZ, and the use of specific siRNA, Kofler et al. were able to characterize a nuclear import and nuclear export sequence within the TAZ molecule itself. Through their investigations, they were able to show that the nuclear localization signal (NLS) is located at the C-terminal end of the TAZ protein, while the nuclear export signal (NES) is located in the TEAD-binding domain. When TAZ is active, it complexes with TEAD, which masks the binding domain, thereby preventing the nuclear export of TAZ. The C-terminal NLS region in TAZ represents a new class of transport motifs, since TAZ, in contrast to the nuclear import mechanism previously described for YAP, does not require binding to Ran. With regard to the mechanobiological regulation of TAZ nucleus–plasma shuttling, Kofler et al. [44] demonstrated that the NLS is dependent on RhoA; i.e., RhoA activity directly stimulates the import of TAZ into the cell nucleus. Intracellularly, the activation of the small GTPase RhoA in response to environmental mechanical cues, like acute tensile stress in epithelial monolayers, yields actomyosin assembly, for instance, at sites of tension-bearing cadherin-based AJs [45].

#### 2.1.2. FAK

**Figure 2:**
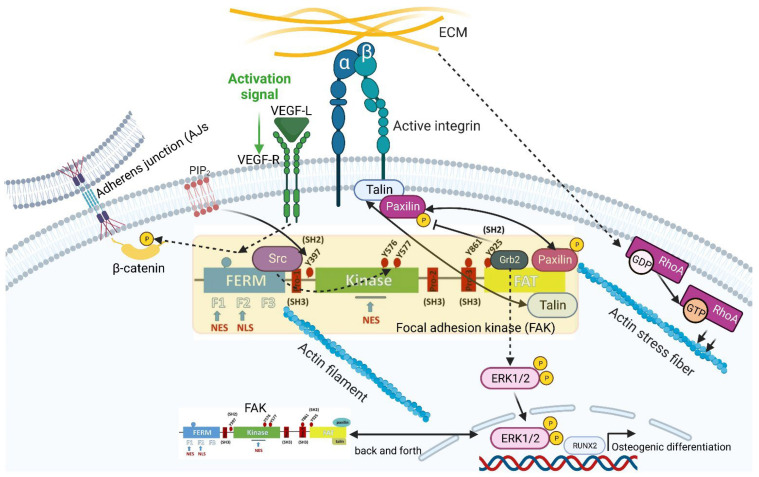
Model of FAK activation mediated by different stimuli. FAs concomitant with actin stress fiber formation have been found to require RhoA activation in response to extracellular mechanical cues. FAK is activated upon integrin engagement. But first, clustering of FAK at the cell membrane lipid bilayer is induced by phosphatidylinositol-4,5-bisphosphate (PIP2) with the prior binding of PIP2 to the FAK basic region of FERM, leading to a partially opened conformation of the FAK molecule and to the exposure of the autophosphorylation site at tyrosine residue 397 (FAKp397). This partially opened conformation of FAK with FAK Y397 leads to the recruitment of Src molecules. These src molecules, in turn, lead to src-dependent phosphorylation of FAK Y576/577 within the kinase domain and prove that PIP2 is key to linking integrin signaling to FAK activation. FAK can also be phosphorylated at tyrosine residue 925 (FAK Y925). This phosphorylation is also carried out by growth-factor-receptor-bound protein 2 (Grb2). Grb2 can lead to the growth-factor-independent activation of the MAP kinase ERK2. Furthermore, the link between FAK and ERK1/2 can trigger osteogenic differentiation via the expression of TF Runt-related transcription factor 2 (RUNX2). Since the dissolution of FAs is putatively related to the phosphorylation of the FA component paxillin, it was found that this, in turn, is regulated by the impaired phosphorylation of FAK Y925. FAK is not only limited to the vertical contact area with the ECM but is also involved in mechanotransduction emerging from horizontally aligned cell–cell contacts such as adherens junctions (AJs). Here, FAK is able to specifically phosphorylate AJ-inherent β-catenin in response to vascular endothelial growth factor (VEGF) treatment. Since FAK can switch back and forth between the nucleus and cytoplasm, FAK has two nuclear export signal (NES) domains and one nuclear localization signal (NLS) domain. The schematic was created with BioRender.com.

In a review that is as empathetic as it is science-enthusiastic, one of the pioneers in identifying and characterizing the molecular building blocks of FAs, Keith Burridge [17], describes their discovery, which was achieved by overlaying electron microscopy (EM) and interference reflection microscopy (IRS) images obtained from cultures from other colleagues in 1978 [46,47]. FAs concomitant with actin stress fiber formation have been found to require RhoA activation in response to extracellular mechanical cues [17] (Figure 2). The fact that various signal cascades emanate from FAs, which are mechanobiological regulators of cell behavior, became particularly clear with the discovery of FAK (focal adhesion kinase), which was independently discovered by three scientists in 1997 [48,49,50]. FAK was identified using a homology-based cDNA cloning approach in chick embryonic fibroblasts as a substrate of the proto-oncogene tyrosine kinase sarcoma, “SRC” for short [50].

In 2014, Goñi et al. [51] succeeded in identifying and characterizing the activation of FAK upon integrin engagement, which takes place in several steps, with the help of a multidisciplinary approach. In the course of their work, the group members found that the clustering of FAK at the cell membrane lipid bilayer was induced by phosphatidylinositol-4,5-bisphosphate (PIP2) (Figure 2). A prerequisite for FAK clustering was the prior binding of PIP2 to a basic region within the FERM (Band 4.1, Ezrin, Radixin, Moesin) regulatory domain of FAK. Within these FAK clusters, PIP2 then induced a partially opened conformation of the FAK molecule, leading to the exposure of the autophosphorylation site at tyrosine residue 397 (FAK^p397^). This initial step favored the further autophosphorylation of FAK molecules and thus the recruitment of src as well. Subsequently, the phosphorylation of the activation loop within FAK by src led to the release of the FERM/kinase tether and full catalytic activation. In this way, the group succeeded in demonstrating that PIP2 is key to linking integrin signaling to FAK activation [51]. Using HeLa cells as an in vitro test system, Goñi et al. used mechanistic functional analyses of FAK signaling in FAs to examine whether PIP2 is actually involved in the autophosphorylation and src-dependent phosphorylation of FAK^p576/577^ within the kinase domain. The pivotal function of PIP2 in this context was confirmed by the knockdown of phosphatidylinositol 4-phosphate 5-kinase type Iγ (PIP5KIγ (isoform 2), the enzyme that catalyzes the formation of PIP2. This knockdown led to a drastic loss of FAK^p397^ and FAK^p576/577^, while the total FAK protein level remained the same [51].

Accumulating evidence indicates that FAK acts as an essential central hub that finely regulates multiple cellular processes, such as cell cycle progression and proliferation, growth, spread and migration, survival, angiogenesis, epithelial-to-mesenchymal transition (EMT), cancer stemness, and the establishment of an immunosuppressive TME (tumor microenvironment) [52,53]. Due to the large number of FAK-regulated mechanobiological processes, which this review cannot do justice, some of them will be explained below using specific tyrosine phosphorylations as examples.

In addition to tyrosine phosphorylation at positions 397 and 576, as well as 577, described above, FAK can also be phosphorylated at tyrosine residue 925 (FAK^p925^) (Figure 2). Members of the src kinase family also carry out this phosphorylation. FAK^p925^ creates a binding site for the src-homology 2 domain (SH2, which mediates protein–protein interactions) of the small signal-transduction-related adapter protein growth factor receptor-bound protein 2 (Grb2). Grb2, along with other intracellular signaling pathways, can lead to the growth-factor-independent activation of the MAP kinase ERK2 in the context of further FAK downstream signaling (Figure 2). Among other functions, ERK-MAP kinase signaling is, in turn, involved in the control of cell differentiation and proliferation. While the molecular link between FAK, ERK1/2, and the osteogenic-differentiation-triggering Runt-related transcription factor 2 (RUNX2) could be demonstrated in human MSCs [54] (Figure 2), the FAK-ERK2 relevance for proliferation was shown in SiHa cells (squamous cell carcinoma cells derived from uterine tissue) by conducting experiments with selective FAK- and MAP-kinase-specific inhibitors [55]. However, using respective FAK mutants that could no longer bind Grb2 in 293T cells, Schlaepfer et al. [56] showed in 1997 that the binding of Grb2 to FAK is not crucial for ERK2 activation mediated by integrin signaling. This finding shows that the activation of ERK2 via Grb2 is just one of many intracellular pathways.

Deramaudt et al. [57] published another function of FAK^p925^ in 2011. Here, using mouse embryonic fibroblasts (MEFs), they found that FAK^p925^ plays an important role in the disassembly of FAs and thus cell migration. By creating mutated FAK MEFs, they could demonstrate that these cells, which express non-phosphorylatable FAK^p925^ (Y925F-FAK), exhibited stabilized FAs, resulting in impaired FA dissolution in conjunction with diminished cell migration [57]. Since the dissolution of FAs is putatively related to the phosphorylation of the FA component paxillin, Deramaudt et al. investigated this functional relationship. Indeed, they found that in Y925F-FAK cells, in which the dissociation kinetics of FAs was severely retarded, paxillin phosphorylation was very low. Conversely, confocal sequential time-lapse microscopy images indicated that the number of stable FAs in Y925F-FAK cells was significantly increased. The speed of forming migration-associated cell protrusions was also significantly slower in Y925F-FAK cells. These results show that the turnover of FAs and thus cell migration are decisively regulated by FAK^p925^ via paxillin [57] (Figure 2).

Although a multitude of mechanobiologically relevant functions have been discovered to be associated with FAs, further research on cell–cell contact structures since the millennium has provided increasing evidence that FAK is not only limited to the vertical contact area with the ECM but is also involved in mechanotransduction emerging from horizontally aligned cell–cell contacts, such as AJs. With regard to this completely new aspect, research by Chen et al. [58] showed that, with the help of kinase assays, recombinant FAK was able to specifically phosphorylate AJ-inherent β-catenin in human umbilical vein endothelial cells (HUVECs) at tyrosine residue (Tyr) 142 in response to vascular endothelial growth factor (VEGF) treatment (Figure 2). Mechanistically, this Tyr 142 phosphorylation enhances (VE)-cadherin-built AJ liquidation and thus vascular permeability by interfering with the binding of α-catenin to β-catenin [58]. These results, reported by Chen et al. [58], revealed two important new aspects of FAK. Firstly, the presence of FAK is not limited to FAs but can also be found on cadherin-mediated AJs, and, therefore, there is FAK-mediated crosstalk between FAs and AJs. Secondly, the findings also show very impressively that FAK cannot exclusively be activated via mechanical–biomechanical extracellular signals, which emerge from FAs or AJs, but rather via biochemical ones as well, in this case, VEGF, which, via its receptor VEGFR, activates its intracellular signaling (Figure 2).

An interesting facet of FAK was discovered in 2008, namely, that FAK is not only an essential component of FAs but also able to migrate back and forth between the cytoplasm and the nucleus [59]. In the same year, in 2008, Lim et al. [60] used MEFs to show that FAK has an NLS in the F2 lobe of the FERM domain (among others, this is discussed in more detail in FAK in Cancer, Section 2.4.2). Moreover, Ossovskaya et al. [59] showed that FAK has two NESs (normally comprising 4-5 hydrophobic amino acid residues [61], e.g., leucine): NES1 within the F1 lobe of the FERM domain and NES2 within the kinase domain (Figure 2). With the help of GFP-FAK-transfected HUVECs, they were able to show that, based on constitutive GFP-FAK nuclear fluorescence, the addition of leptomycin B, a nuclear export inhibitor, prevented FAK from leaving the nucleus. From this result, Ossovskaya et al. [59] concluded that FAK must have NESs. Using differential transfection construct combinations of NES1 and NES2 with the NLS (correspondingly, NES1-NES1-NLS and NES2-NES2-NLS), the same group was able to demonstrate in MEFs that NES2 has a stronger potential than NES1 to transport FAK out of the cell nucleus.

Because FAK can switch back and forth between the nucleus and cytoplasm, it is possible for FAK to modulate gene expression by affecting the expression of TFs. Hence, it is of note that FAK has an active nuclear import signal and can enter the nucleus. Whether there are active mechanisms shuttling FAK to the nucleus or whether certain cellular states increase the capacity of FAK interactors in the nucleus to retain FAK is currently unknown. Interestingly, one stimulus resulting in increased FAK levels in the nucleus is mechanical force [62]. An example affecting downstream TF expression is the binding of p53 to the FERM domain and its later degradation. p53, as a TF, regulates the expression of p21, an inhibitor of cell-cycle-promoting cyclin-dependent kinases (CDKs). Therefore, the loss of nuclear-FAK-driven loss of p53/p21-mediated cell cycle control can favor carcinogenesis, an issue that will be discussed in more detail in Section 2.4.2 (see also Lim et al. [60]). Another example of FAK interaction with a TF is myocyte enhancer factor 2 (MEF2), a master cardiac transcriptional regulator. In contrast to p53, however, MEF2 does not bind to FERM but rather to the focal adhesion targeting (FAT) domain (the FAT domain also interacts with, e.g., the FA constituents paxillin and talin as part of the FAK scaffolding function). FAK, as a co-transcriptional activator in complex with MEF2, can form a ternary complex with DNA and thereby controls the expression of the cellular jun-gene-encoded TF (c-Jun, in combination with protein c-Fos, forms the AP-1 early response transcription factor), as demonstrated by Cardoso et al. [62]. By using the cardiomyoblast H9c2 cell line as an in vitro model system of heart cells, Cardoso et at. [62] further showed that FAK signaling is important for MEF2 activity. In H9c2 cells, overexpression of wild-type FAK yielded the strong expression of the MEF2-responsive reporter, while mutant FAK failed to do so. Further experiments using a MEF2-responsive Jun promotor reporter gene approach in cultured neonatal rat ventricular myocytes (NRVMs) revealed that although MEF2 alone is capable of inducing the c-Jun promotor, the combined action of the FAK/MEF2 complex exhibited remarkable potentiation of the MEF2-related induction of c-Jun expression [62,63]. These findings demonstrate that nuclear FAK, like the aforementioned key mechanobiological player molecules YAP and TAZ, can not only act as a co-transcriptional activator but also participate in the regulation of TF gene expression. Against this background, an exciting and, therefore, open research area is to identify further nuclear FAK interaction partners, an issue that also holds true for YAP/TAZ, regarding the regulation of genes and thus the expression of proteins.

#### 2.1.3. Short Summary

Taken together, YAP/TAZ are biomechanically sensitive co-transcription activators, whose biological function is mutually regulated by the biomechanics of the extracellular environment, on the one hand, and the biomechanics of the cell, on the other. In the course of this reciprocity, they contribute to the control of almost all cell behavioral expressions of cells in physiological and pathophysiological cell and tissue states. In this context, recent work has been able to show how the interaction of YAP/TAZ with preferential TFs takes place at a mechanistic level and how YAP/TAZ enter and exit the nucleus to fulfill their function as shuttling mechanotransducers.

FAK is involved in the regulation of the turnover of both FAs and AJs. In addition, like YAP/TAZ, FAK acts as a co-transcriptional activator in the cell nucleus through its contribution to the control of TFs on both the protein and gene expression levels (e.g., p53, see Section 2.4.2, FAK in Cancer, and c-jun). Thus, FAK fulfills multiple functions within cells, depending on its subcellular localization. Therefore, based on the definition of a moonlighting protein, FAK may be considered a moonlighting protein as well.

### 2.2. In Vitro Models as Valuable Tools to Elucidate the Role of YAP/TAZ and FAK in Aging and Senescence

#### 2.2.1. YAP/TAZ

In addition to the molecular mechanisms underlying the function of YAP and TAZ as force-dependent shuttling mechanotransducers, there has recently been increasing evidence that these two molecules also play an important role in the cellular aging process, which also involves cellular senescence. Senescence, as a central hallmark of aging, on the molecular level, is characterized by a steady state of cell cycle arrest, which is preferentially induced by cellular stress, DNA damage, and telomer erosion and accompanied by inflammatory secretory cell phenotypes [64,65,66]. Biomarkers that are recognized as indicating senescence include not only the cell-cycle-affecting kinase inhibitors p16, p21, p27, and p53 but also nuclear lamins, such as lamins B1, A, and C, among others [66,67,68,69,70].

**Figure 3:**
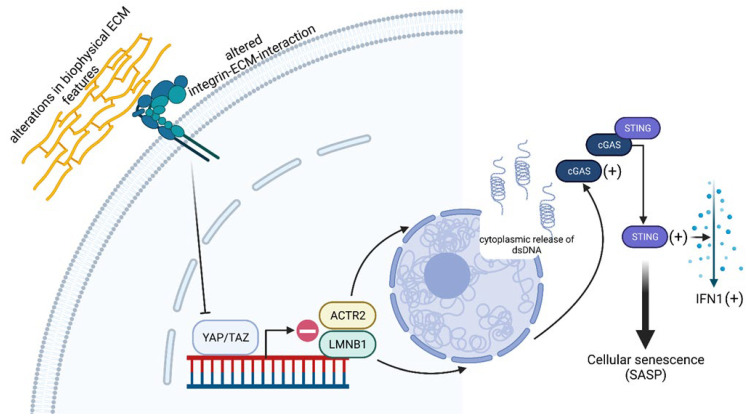
Schematic illustration of YAP/TAZ in aging and senescence. Studies revealed that alterations in biophysical ECM features and integrin–ECM interactions may lead to altered mechanotransduction and reduced YAP/TAZ activity. This YAP deficiency induces the senescence-associated secretory phenotype (SASP), which is based on the secretion of pro-inflammatory molecules and creates an inflammatory cell environment with the involvement of the cyclic GMP-AMP synthase–stimulator of interferon genes (cGAS-STING) signaling cascade. STING thereby functions as a sensor for cytosolic DNA, which binds to and is activated by cGAS. Following its activation, STING induces the synthesis of interferon type I (IFNI) and contributes to the formation of the SASP phenotype. Lamin B1 (LMNB1) is important for nuclear integrity and has been discovered as a reliable marker of senescence. There is a direct connection between YAP/TAZ and LMNB1 since this lamin was almost undetectable in YAP/TAZ-depleted cells. ARPC2/3, which codes for the actin-related protein 2/3 complex (ACTR2 = ARPC2/3) involved in actin polymerization, was also downregulated in YAP/TAZ-depleted cells, and this decrease in ARPC2/3 led to a loss of the actin cap with the consequent bulging of the nuclear envelope and its increasing wrinkling and cytosolic DNA release. This means that ARPC2/3 and LMNB1 are further target genes of YAP/TAZ. The schematic was created with BioRender.com.

A very important published finding in this context is that a progressive decline in mechanobiologically relevant YAP/TAZ functioning goes hand in hand with a functional and structural decline in aging tissues. This relation was recently published by Sladitschek-Martens et al. [71], who combined in vivo approaches in mice with in vitro experiments using cells of different tissue origins as model systems. Among these, in addition to human fibroblasts (WI-38), primary dermal fibroblasts from adult and young mice (MAFs), as well as primary mouse aortic smooth muscle cells (SMCs) and human embryonic kidney (293T) cells, were employed. Although the basis of the decline in YAP/TAZ function still needs to be fully elucidated, Sladitschek-Martens et al. [71] discuss environmental as well as cell-intrinsic changes as causatives. In detail, alterations in (i) biophysical ECM features, (ii) viscoelastic tissue properties, (iii) integrin–ECM interactions, or (iv) actin-change-related defective cell contractility (see also Section 2.2.2) were hypothesized, which may lead to altered mechanotransduction and reduced YAP/TAZ activity. In their studies, they were able to show that the depletion of YAP/TAZ in old fibroblasts increased the activity of β-galactosidase, a classic senescence marker, an effect that could be reverted by maintaining YAP/TAZ function experimentally. Furthermore, cytokine arrays could show that YAP deficiency induces the senescence-associated secretory phenotype (SASP) [71] (Figure 3), which is based on the secretion of pro-inflammatory molecules and creates an inflammatory cell environment [72]. Since there is evidence that the cyclic GMP-AMP synthase–stimulator of interferon genes (cGAS-STING) signaling cascade is involved in triggering the SASP phenotype [73], Sladitschek-Martens et al. [71] investigated this connection in more detail. Regarding cGAS-STING, the immune system uses this signaling pathway to detect the presence of cytosolic DNA [74] (Figure 3), which often represents a sign of host damage in response to host invasion by pathogens. Moreover, in cultures of human dental pulp cells (HDPCs), Tian et al. [75] showed that cGAS-STING is an inducer of inflammation, since the knockdown of cGAS-STING abolished the production of inflammation-related cytokines in response to HDPC transfection with bacterial DNA, suggesting its important role in host defense. With respect to the detailed analysis, Sladitschek-Martens et al. [71] detected cGAS accumulation at the interface of the cytoplasm and the nucleus and at sites of nuclear fractures in the cytoplasm in primary fibroblasts following YAP/TAZ depletion. To place these observations in a mechanobiological context, the same group cultured fibroblasts on soft hydrogels, thereby reducing the tensile forces of the extracellular environment. This reduction resulted in the inactivation of YAP/TAZ and, in turn, the activation of cGAS, demonstrating that mechanical cues are involved in the regulation of the YAP/TAZ signaling axis (Figure 3). In terms of these results, the situation was comparable when the cells were cultured on small areas and acquired a rounded morphology, a phenomenon also observed in senescent fibroblasts [76]. However, cGAS activation was reverted upon the reactivation of YAP, thus pointing to the reversibility of the process. Regarding the adapter molecule STING, it is located at the endoplasmic reticulum and functions as a sensor for cytosolic DNA, which binds to and is activated by cGAS. After its activation, STING induces the synthesis of interferon type I (IFNI), which is why it plays an important role in inflammation and thus also contributes to the formation of the above-described SASP phenotype [73,74] (Figure 3). Under in vitro conditions, Sladitschek-Martens et al. [71] were able to show that SASP-associated genes and characteristic type-I-interferon-regulated genes were activated in human fibroblasts as a result of RNAi-mediated YAP/TAZ inactivation. From this and other findings, it was concluded that STING is the driver of YAP/TAZ-loss-induced effects. From the previously described finding of cGAS accumulation at sites of nuclear discontinuities, it was hypothesized that YAP/TAZ are involved in maintaining nuclear integrity, and that changes in the shape of the nucleus may be related to age-related senescence, since such changes have already been observed in senescent fibroblasts in vitro. In a very recent study, Heckenbach et al. [77] were able to show in three established human-derived primary skin fibroblast cell lines (HPSFs), where senescence was induced by ionizing radiation, that nuclear morphology is an accurate and, therefore, reliable senescence predictor in cultured cells. In this context, it is important to note that, under in vitro conditions, the nuclear shape of mouse fibroblasts is intimately connected to a dome-like arrangement of actin filaments, the so-called perinuclear actin cap, which encases the apical surface of the nucleus and thus protects it from deformation [78,79]. In young mouse fibroblasts, this actin cap is lost when YAP/TAZ are depleted, and it is absent in old fibroblasts. The fact that YAP/TAZ plays a mechanistic role in the maintenance of the actin cap was demonstrated by the restoration of YAP in aged cells [71]. In search of the basis of this mechanistic role of YAP/TAZ, Sladitschek-Martens et al. [71] screened YAP/TAZ target genes that may be related to nuclear integrity. Within the group of identified candidate genes, the initial focus was on lamin B1 (LMNB1), a nuclear lamina structural protein involved in nuclear integrity. The role of LMNB1 in nuclear integrity has been previously validated by an in vitro cell system based on the human osteosarcoma cell line U2OS, which showed a 60% reduced expression level of LMNB1 (U2OS GFP-NLS shLmnB1) and a significantly higher tendency of spontaneous rupture of the nuclear envelope [80,81,82]. In addition, it could be shown that the induction of senescence in primary and murine cells led to a loss of LMNB1 expression, which qualifies LMNB1 as a reliable marker of senescence [70] (Figure 3). With reference to LMNB1, Sladitschek-Martens et al. [71] were able to show, in their current study, the existence of a direct connection between YAP/TAZ and LMNB1. Here, this lamin was almost undetectable in a human-lung-fibroblast-based cell system (WI-38) and adult mouse fibroblasts because of YAP/TAZ depletion at both the mRNA and protein levels [71]. To identify additional molecules associated with nuclear integrity, Sladitschek-Martens et al. [71] downregulated the expression of all found candidate genes by RNA interference (RNAi) and screened for the expression of cysteine-x-cysteine (C-X-C) motif chemokine ligand 10 (CXCL10), also known as interferon-gamma-induced protein 10 (see the connection with STING-IFNI mentioned below). The gene ACTR2 was identified, which codes for the actin-related protein 2/3 complex (ARPC2/3) involved in actin polymerization within the ARPC2/3 complex. Further investigations revealed that ARPC2/3 was also downregulated in YAP/TAZ-depleted cells. In addition, a decrease in ARPC2/3 in fibroblasts led to a loss of the actin cap with the consequent bulging of the nuclear envelope and its increasing wrinkling. These developments coincided with the induction of cGAS-STING-regulated SASP-associated genes. Subsequent experiments, such as chromatin immunoprecipitation in fibroblasts and vascular smooth muscle cells, have provided increasing evidence that ARPC2/3 and LMNB1 are further target genes of YAP/TAZ [71] (Figure 3). The results of this most recent study on the role of YAP/TAZ in mechanobiology show its involvement in senescence-associated cellular aging processes, but this has not yet been fully characterized, and, therefore, this makes these molecules attractive for gerontology in the future. Since Sladitschek-Martens et al. [71] preferentially focused on tissues and cells of mesodermal/mesenchymal origin, like the dermis or fibroblasts and smooth muscle cells, another open research question is whether the impact of YAP/TAZ on the control of senescence is a general phenomenon, meaning that it applies to all cell entities of the human body, or whether it is tissue- and, therefore, cell-type-specific. The molecular relationships underlying how the mechanosignaling of YAP/TAZ contributes to the aging process are shown as a model in Figure 3.

#### 2.2.2. FAK

If one looks at the specialist literature on senescence between the years 2004 and 2022, an ambivalent picture emerges regarding the role of FAK in the context of senescence. This means that there is evidence that the activity of FAK, mainly the phosphorylation at tyrosine residues 397, 576, and 577, can be both pro- and anti-senescent.

Looking at the aging- and senescence-related studies published in this period in chronological order, Cho et al. [83] used diploid human foreskin fibroblasts (HDFs), young versus old, as an in vitro cell system and described the flattening of the cells as a morphological change in the context of cell aging and senescence. At the molecular level, this flattening was associated with an increase in actin stress fibers and the FA constituents integrin β1, FAK, and caveolin-1 (CAV1). As the main constituent of small plasma membrane invaginations (caveolae), CAV1 can interact with many signaling molecules, thereby, for instance, inhibiting not only EGF receptor signaling-induced proliferation but also migration, as shown for the human colorectal cancer cell line CRC (SW480) [84]. With respect to FAK and paxillin, higher levels of phosphorylation were detected in senescent cells, from which Cho et al. [83] concluded an increased number of FAs. By using the RNAi approach, it could be shown mechanistically that CAV1 is involved in the maintenance of the senescence-indicating flattened cell morphology and downmodulation of CAV1, which corresponded to significantly reduced FAK activity (detected by general FAK phosphorylation FAK^p^). In addition, the disruption of stress fiber formation was observed as a further consequence of CAV1 intervention [83]. Hence, the results reported by Cho et al. [83] present evidence that FAK activity is pro-senescent.

In a study published in 2005 by Nishio and Inoue [85] comparing fetal lung fibroblasts (WI138-VA13) with senescent human adult skin fibroblasts (TIG101), it was found that the senescent phenotype with a flattened morphology and predominantly thin instead of thick actin fibers showed a reduction in the expression of the non-receptor tyrosine kinase c-src (SRC). Although no direct experiments were carried out on the phosphorylation status (activity) of FAK, the authors indirectly concluded from the findings of the senescence-related reduction in SRC expression that FAK was also hypo-phosphorylated in the senescent cells [85]. From this, it follows that reduced FAK activity supports senescence and that active FAK thus has an anti-senescent effect.

An analysis of aging and the contractility of muscle-supplying arteries in rats revealed that rat-derived vascular smooth muscle cells (VSM) from older animals showed reduced FAK activity (FAK^p397^) compared to younger animals. This reduced FAK activity was accompanied by a switch from alpha smooth muscle actin (ACTA2) to gamma SMA (ACTG2) in the VSM cells of old animals, with the ACTA2/ATCG2 shift leading to increased cell stiffness and thus reduced vascular contractility [86]. These results provide evidence for an anti-aging effect of FAK activity.

Using an ATP-competitive FAK inhibitor, Alza et al. [67] were able to show that the inhibition of FAK activation (FAK^p397^) led to a proliferation arrest and, in addition to the increase in the number of β-galactosidase-positive cells, an increase in cell size, i.e., visible and measurable attributes of senescence. At the molecular level, FAK inhibition resulted in increased levels of p27, an inhibitor of cell-cycle-dependent kinases (CDKs; see also Section 2.2.1), in glioblastoma cell lines (U251-MG and 487-MG). Furthermore, Alza et al. [67] demonstrated in their experiments that FAK inhibition led to a decrease in p62, an autophagy-relevant molecule, which can act as a cargo receptor and autophagy substrate as well. By inhibiting p62 expression using RNAi, Alza et al. [67] proved that this p62 reduction is causally involved in senescence and the associated proliferation arrest. This was indicated by an increased amount of β-galactosidase activity concomitant with an elevated level of the proliferation-inhibiting p27, which suggests that the CDK inhibitor is regulated by p62 [67]. Based on these findings, the activity of FAK can be considered anti-senescent.

As already discussed in the previous section, Section 2.2.1 on YAP/TAZ, the integrity of the cell nucleus plays an important role in senescence. In this context, Chuang et al. [69] showed that, in cell systems comprising non-small lung cancer cell lines A549 and H1299, the inhibition of FAK’s catalytic function (FAK^p576, 577^) resulted in the downregulation of the nuclear matrix proteins lamin A and C, which was associated with nuclear deformation. In coincidence with the findings for YAP/TAZ, these results emphasize the role of lamins and thus nuclear integrity in counteracting senescence-related nuclear deformation. Furthermore, the inhibition of FAK activity led to an increase in β-galactosidase activity and an increase in p53 (see also Section 2.2.1) expression [69]. Within the relationship between FAK and p53, and especially the role of p53 in senescence, at the mechanistic level, p53 acts as a master regulator of the cell cycle via the upregulation of CDK inhibitors. This is exemplified by p21 or p27. They can induce cell cycle arrest, which is a typical sign of senescence. On the other hand, p53 can also induce apoptosis, for example, by upregulating pro-apoptotic BH3-only proteins that bind to anti-apoptotic proteins. For this reason, cells in the senescent stage limit the activity of p53. MDM2, the mouse double minute 2 homologous molecule (also known as E3 ubiquitin-protein ligase), which is involved in the proteasomal degradation of p53 (see also Section 2.4.2), should be mentioned here as an example [87]. In this context, the already-mentioned study by Nishio and Inoue [85] is interesting, since they were able to detect an abundance of cytoplasmic vimentin (intermediate filament)-bound p53, thus indicating the relationship between FAK and p53. Nishio and Inoue [85] interpret this finding to mean that it may also represent a mechanism by which senescent cells regulate p53 activity.

In a recently published study, which expands on the previously described work by Chuang et al. from 2019 [69], the authors were able to show that the inhibition of FAK (FAK^p397^) leads to a reduction in the expression of the histone methyltransferase enhancer of zeste homolog 2 (EZH2) [68]. EZH2 is involved in histone 3 (H3) methylation at lysine residues 9 and 27, leading to transcriptional repression, which, in turn, promotes proliferation [88]. Ectopic expression of EZH2 attenuated senescence in FAK-inhibited cells, leading the authors to conclude that EZH2, which is active in many tumors, is a downstream target of FAK [68]. Both studies by Chuang et al. from 2019 and from 2022 show that the activity of FAK has an anti-senescent effect [68,69].

Another study that shows the pro-senescent effect of FAK activity was published by Shin et al. [66] in 2020. Here, the observation that βPIX (ARHGEF7), which is an FA-localized guanine nucleotide exchange factor, is downregulated during the aging process in vivo and in in vitro cell systems led to further investigations in the context of senescence. First, the authors were able to detect the anti-proliferative CDK inhibitor p16 in the tissues of aged mice and human diploid fibroblasts (HDFs); following ARHGEF7-RNAi application, p16 was also upregulated within the course of senescence (see also Section 2.2.1). In addition, the ARHGEF7-interacting exchange factor (a Cdc42/Rac1-activated kinase), which activates the small GTPases Rac and Rho, is involved in FA turnover and cell migration [89]. Furthermore, ARHGEF7 depletion in HDFs led to an increase in FAK activity (FAK^p397/576^), which was associated with an increased number of focal contacts and the reorganization of actin stress fibers, together with higher numbers of β-galactosidase-positive cells. The inhibition of FAK activity reverted senescence not only in ARHGEF7-depleted HDFs (a pharmacological inhibitor) but also in ARHGEF7-depleted mice (ARHGEF7 RNAi/FAK RNAi, abolishment of senescence indicated by, e.g., loss of p16), indicating the functional involvement of FAK activity in senescence. The same reversion of senescence was observed when the ARHGEF7 -/- HDFs were cultured in the presence of FA–integrin-antagonizing RGD peptides, a finding from which the authors concluded that ARHGEF7 reduction yields altered integrin signaling, which, in turn, promotes FAK activation and senescence [66].

#### 2.2.3. Short Summary

Taken together, in connection with aging and senescence, the evidence that YAP/TAZ can also play a major role appears to be supported by brand-new studies. Here, molecules of inflammation, such as cGAS-STING, are combined with the cellular depletion of YAP and the biomechanics of the extracellular environment, in this case, a soft one. Subsequent analyses could show that, in the context of inflammation, cGAS-STING-related YAP/TAZ loss promotes the SASP phenotype and further leads to a loss of nuclear integrity, typical of senescent cells. This is because YAP/TAZ appears to be causally involved in the transcription of nuclear stabilizing molecules such as ARPC2/3 and LMNB1.

With respect to FAK, the examples presented show the complexity of its role in senescence and thus the aging process as well. Since the in vitro cell systems used are cells from (i) different species, (ii) different tissues, and (iii) different cell transformation stages, i.e., how far the cells have progressed in the multi-stage process of carcinogenesis, all three factors can contribute to the ambivalent role of FAK in senescence. This results in an open research question that still needs to be addressed: which of the factors mentioned influence senescence with FAK as the target molecule, and in what way? The ambivalence of FAK in the context of senescence described at the beginning of this subsection also raises the question of whether therapeutic intervention for FAK (discussed in detail in Section 2.5.2) is always the method of choice. This is because there is evidence that senescence, even though it sends cancer cells into irreversible cell cycle arrest, can also exert pro-oncogenic effects due to the SASP. This aspect is exemplified by skin tissue, in which the SASP phenotype of senescent cells contributes to the promotion of skin carcinogenesis through mitogen-activated protein 38 (p38MAP) kinase (MAPK) and p44/42 MAPK signaling [90]. Moreover, the SASP issue, which should be examined more closely by further investigations, also represents a scientific and clinical challenge in the context of unresolved research questions.

### 2.3. Cell Systems to Investigate YAP/TAZ and FAK in Wound Healing

#### 2.3.1. YAP/TAZ

During wound healing, the destroyed ECM must be restored, which means that different biomechanical ECM stiffnesses act on the cells involved in wound healing, such as connective tissue fibroblasts, myofibroblasts, and epithelial cells. As the previous sections have made clear, such stiffness changes yield causal changes in mechanotransduction and thus cell behavior as well. For this reason, the participation of YAP and TAZ as force-sensitive mechanotransducers in wound healing is discussed below.

**Figure 4:**
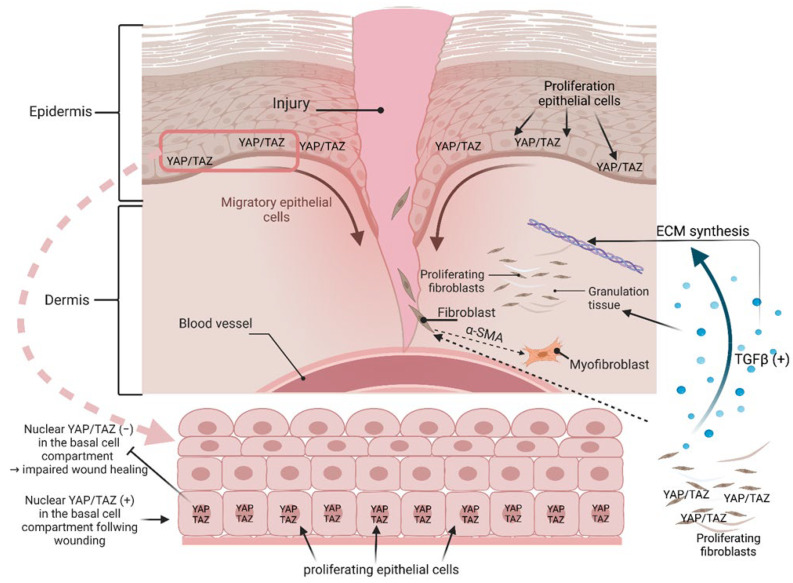
Schematic illustration of YAP/TAZ in wound healing. It is known from human skin that YAP/TAZ have a nuclear localization predominantly in the basal cell layer, which indicates their bioactivity as co-transcription activators in the basal cell region. After wounding, YAP/TAZ were also found to be increased in the nucleus, preferentially in basal cells. YAP/TAZ in fibroblasts supported the notion that both molecules are required for the expression of TGFβ/TGFB1, which could be shown to promote the formation and organization of granulation tissue and ECM synthesis, as well as the conversion of dermal fibroblast to myofibroblasts by the induction of α-smooth muscle actin (α–SMA, ACTA2). The schematic was created with BioRender.com.

Skin wound healing. As the skin is considered the largest organ in humans, it is an attractive target to investigate the role of YAP and TAZ in the context of tissue homeostasis and regenerative tissue conditions, like wound healing. From immunohistochemistry on murine and human skin, it is known that YAP/TAZ have a nuclear localization predominantly in the basal cell layer, which indicates their bioactivity as co-transcription activators in the basal cell region (Figure 4). In double-knockout (dKO) mice, i.e., mice with simultaneous KO of YAP and TAZ, the number of proliferative cells in the basal cell compartment decreased significantly, which can be interpreted as an indication of the involvement of YAP/TAZ in epidermal tissue homeostasis [91]. After wounding the control mouse skin, YAP/TAZ were also found to be increased in the nucleus, predominantly in basal cells, which suggests their key role in wound healing (Figure 4). This observation was again supported by KO mice, in which wound healing and wound closure were delayed after skin injury, accompanied by a significantly reduced number of proliferating basal cells. Moreover, YAP/TAZ were detected in the dermis of healing wounds and were markedly decreased in response to RNAi [91,92]. With respect to the underlying mechanisms of wound healing, the KO of YAP/TAZ in NIH3T3 fibroblasts supported the notion that both molecules are required for the expression of TGFβ (YAP and TAZ regulate skin wound healing, TGFB1) [92]. This could be shown by the formation and organization of granulation tissue and ECM synthesis [93] as well as the conversion of dermal fibroblast to myofibroblasts by the induction of α-smooth muscle actin (ACTA2) [94] (Figure 4). Another facet of how YAP can contribute to wound healing comes from studies on spiny mice and their dermal fibroblasts. Compared to normal mice, spiny mice show no scarring during wound healing, and the subcellular localization of YAP seems to be causally responsible for this. In this context, it could be shown that, under in vitro conditions, human dermal fibroblasts (DFs) and dermal fibroblasts of mice are sensitive to the TGFB1-induced conversion to myofibroblasts. Interestingly, spiny mouse dermal fibroblasts are insensitive, and this TGFB1 insensitivity correlated with the nuclear abundance of YAP, which was absent in the other fibroblast entities. From a mechanistic point of view, it could be shown, by using appropriate enzyme inhibitors, that the YAP/TAZ-dephosphorylating protein phosphatase 2A (PP2A), known to activate YAP [95], was responsible for the nuclear persistence of YAP in the cell nucleus in DFs from spiny mice. This constitutive enzyme activity apparently represents an adaptive mechanism toward cell-surrounding fibrokines (fibrosis-triggering signals), such as TGFB1, which is associated with the absence of scars in spiny mice [96]. Another facet of YAP/TAZ’s roles in inflammation and wound healing is the involvement of gp130, a co-receptor for IL-6 cytokines, which triggers the activation of YAP and Notch, transcriptional regulators that control tissue growth. This signaling module is strongly activated upon mucosal injury to promote healing and maintain barrier function [97].

**Figure 5:**
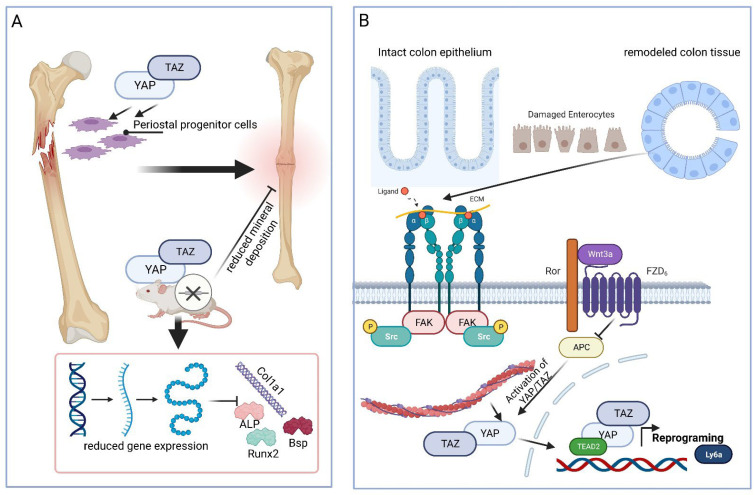
Schematic illustration of YAP/TAZ in bone regeneration (**A**) and recovery from ischemia–reperfusion injury (IRI) (**B**). YAP/TAZ is involved in bone fracture regeneration situations. Mineral deposition was reduced following YAP/TAZ deletion, and the expression of osteogenic differentiation markers, such as Runx2, collagen 1a1 (Col1a1, COL1A1), bone sialoprotein (Bsp, IBSP), and alkaline phosphatase (ALP, ALPL), was reduced. This is important since, in response to bone fracture, periosteal progenitor cells, after proliferation and expansion, differentiate to form cartilage and bone within the fracture callus. In the healing colon epithelium, YAP/TAZ have been shown to promote colon cell reprogramming. Here, β1-integrin-mediated FAK signaling is typical of the healing of epithelia and important for the activation of YAP. Knockout of Apc led to YAP no longer being inactivated, and the cells started growing. The activity of YAP correlated not only with cell growth but also with the expression of fetal markers, such as the lymphocyte antigen 6 gene family member Ly6a, which characterize the healing murine colon epithelium during reprogramming. The schematic was created with BioRender.com.

Bone fracture healing. Bone fracture is another example of the significant involvement of YAP and TAZ in regenerative tissue situations. Among other things that could be shown in vitro in zinc-finger-containing osteoblast-specific TF (SP7)-expressing periosteal progenitor cells was that the mineral deposition was reduced following YAP/TAZ deletion (Figure 5A). As a further consequence of YAP and TAZ deletion, the reduced expression of osteogenic differentiation markers such as Runx2, collagen 1 (COL1A1), integrin binding sialoprotein (IBSP), and alkaline phosphatase (ALPL) could be detected. This impairment in expressing osteogenic differentiation markers is an important point since, in response to bone fracture, periosteal progenitor cells, after proliferation and expansion, differentiate to form cartilage and bone within the fracture callus [98] (Figure 5A).

Colon epithelium healing. In the healing colon epithelium, YAP/TAZ have been shown to promote colon cell reprogramming to the primitive/fetal stem cell phenotype, with the latter being essential for tissue regeneration in response to injury. To analyze this prerequisite in more detail, a 3D organoid model of mouse colon epithelial cells was used, which consisted of collagen(s) type I and type IV as an extracellular environment to induce β1-integrin-mediated FAK signaling. The latter is typical of a healing epithelium and important for the activation of YAP [99] (Figure 5B). In this in vitro culture system, the gene knockout of the β-catenin destruction complex constituent adenomatosis polyposis coli gene (APC) led to YAP no longer being inactivated during capture within the destruction complex [100], but instead, it was activated and the cells in the organoid proliferated. The activity of YAP is correlated not only with cell growth but also with the expression of fetal markers, such as the lymphocyte antigen 6 gene family member Ly6a/stem cell antigen-1 (Sca-1; for a review, see [101]), which characterize the healing murine colon epithelium during reprogramming (Figure 5B). The causal involvement of YAP in fetal phenotype reprogramming was demonstrated by the double knockout of YAP/TAZ, which resulted in growth inhibition, whereas YAP overexpression led to the upregulation of fetal phenotype markers. These findings suggest that YAP is also of great importance in the regeneration of the colon epithelium [99].

**Figure 6:**
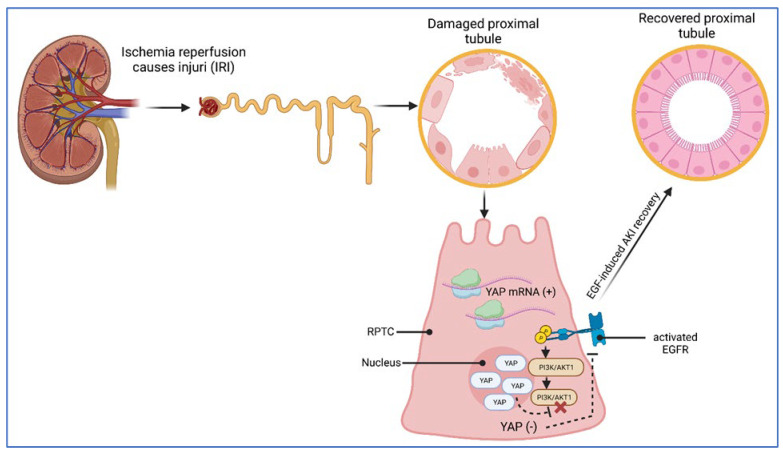
Schematic illustration of YAP/TAZ in ischemia–reperfusion injury (IRI). Renal proximal tubular epithelial cells (RPTCs) isolated from renal mouse tissue displayed elevated levels of YAP mRNA in response to IRI. The same could be detected at the protein level in an IRI model system, in which YAP appeared predominantly with nuclear subcellular localization. Recovery from IRI-induced cell damage obviously requires the activation of the epidermal growth factor receptor (EGFR), which functions as an inductor of phosphatidylinositol 3 kinase (PI3K) protein signaling. Inhibition of EGFR and AKT1/PI3K revealed decreased levels of nuclear YAP and suggested EGFR-AKT1 signaling as a trigger of YAP activation. The schematic was created with BioRender.com.

Ischemia-related acute kidney injury. AKI, formerly acute renal failure (ARF), often occurs during intensive care. Ischemia–reperfusion injury (IRI) causes injury, particularly in renal proximal tubular epithelial cells (RPTCs) (Figure 6). RPTCs isolated from renal mouse tissue displayed elevated levels of YAP mRNA in response to IRI. The same could be detected at the protein level in an IRI model system, in which YAP appeared predominantly with nuclear subcellular localization (Figure 6). In contrast, RPTCs of non-stressed mice showed only a low abundance of nuclear YAP. To mimic IRI under in vitro conditions, RPTCs were cultured in a hypoxic environment, followed by re-oxygenation, thereby leading to YAP nuclear translocation. As demonstrated earlier by Chen et al. [102], recovery from AKI-induced cell damage obviously requires the EGFR-PI3K-AKT1-dependent activation of YAP (Figure 6). Therefore, in a more recent study, Chen et al. [102] inhibited the EGFR in cultured hRPTCs by RNAi or specific drugs and detected decreased levels of nuclear YAP under the hypoxia–re-oxygenation regimen. In addition, decreased levels of nuclear YAP were also observed in hRPTCs transfected with AKT1-specific siRNA. These findings suggest EGFR-AKT1 signaling as a trigger of YAP activation in hRPTCs (Figure 6). On the cell behavioral level, YAP-directed RNAi revealed that YAP activation seems to promote hRPTC proliferation via the upregulation of cyclin D together with amphiregulin (a ligand of the EGFR) and an increase in the phosphorylated state of the retinoblastoma (Rb) protein, with the latter important for the G1-to-S-phase transition within the cell cycle. Based on these mechanistic results, the authors concluded that EGFR-PI3K-AKT1-dependent YAP activation plays an essential role in mediating epithelial cell regeneration during kidney recovery from AKI [102]. The discussed YAP/TAZ involvement in the regeneration of different tissues is schematically depicted in Figure 6.

#### 2.3.2. FAK

To better understand the role of FAK in wound healing, it is helpful to review the stages of this complex process. After (i) the hemostatic phase are (ii) the inflammatory phase, (iii) the proliferation phase, which overlaps with the inflammatory phase, and (iv) the remodeling phase. In particular, the temporal control of the second phase, i.e., the inflammatory phase, plays an important role since a persistent inflammatory environment, characterized by cytokines and chemokines, such as monocyte chemoattractant chemokine-1 (MCP-1/CCL2), leads to fibrosis. It is worth mentioning that in an in vitro vascular model from human aortic endothelial cells, it could be shown with the help of inhibition experiments that, among other things, the activity of FAK is required for the expression of MCP-1. At the molecular–mechanistic level, the MAP kinases p44/42 (ERK1/2) seem to be involved, since the inhibition of FAK together with the downregulation of MCP-1 led to a simultaneous reduction in ERK1/2 activation (phosphorylation) [103]. The dependence of MCP-1 expression on FAK was already shown in an earlier study, in which murine dermal FAK-knockdown fibroblasts showed reduced MCP-1 expression compared to control cells, in which FAK (FAK^p397^) activity was inhibited through a small-molecule approach [104]. In order to simplify the complexity of the wound-healing process, the effect of MCP-1 is regarded as an example. MCP-1 can stimulate proliferation [105] and collagen synthesis in fibroblasts and promote the expression of fibroblast-inherent TGFB1. In turn, TGFB1 can induce the production of ECM and leads to the expression of ACTA2, the typical molecule of myofibroblasts [106]. TGFB1 is considered to be a key inducer of myofibroblast maturation. Interestingly, the expression of integrin αvβ5 (ITGAV/ITGB5) accompanies the transition of fibroblasts to myofibroblasts, and αvβ5 integrin contributes to the conversion of extracellular TGFB1 from the latent to the biologically active form [107]. The findings from Campbell et al. [107] were obtained by employing an in vitro cell system of TGFB1 reporter cells, named TMLCs, which are a stable subclone of mink lung epithelial cells (MLECs). MLECs are stably transfected with an expression construct containing a TGFB1-specific promoter fragment consisting of a truncated plasminogen activator inhibitor-1 (PAI-1/SERPINE1) promoter, which was fused to the firefly luciferase reporter gene [108]. In this way, MCP-1, which is constitutively present in the inflammatory milieu, can contribute to a cycle, so to speak, via TGFB1, collagen synthesis, integrin avβ5, and converted TGFB1, which promotes undesired permanent myofibroblast maturation and thus an oversupply of ECM collagen. This oversupply ultimately causes matrix stiffening in fibrosis, concomitant with altered cellular (fibroblast) mechanotransduction. This alteration in ECM stiffness and thus mechanotransduction leads to the ongoing adverse activation of FAK and FAK signaling in the course of fibrosis [109].

The importance of fibrosis caused by a persistent inflammatory state in the body can be illustrated, for example, by heart failure and, here, in particular, heart failure with a preserved ejection fraction (HFpEF). In HFpEF, an inflammatory comorbidity of, for example, obesity or diabetes, in combination with a hemodynamic load caused by arterial hypertension, leads to the conversion of inflammation- and hemodynamic-load-sensitive myocardial fibroblasts to myofibroblasts, which then trigger fibrosis. Fibrosis, i.e., the overproduction-related stiffening of the ECM within the left ventricle (LV), is one of the critical determinants of myocardial stiffness often observed in HFpEF, with the latter representing a growing public health problem with substantial morbidity and mortality [110,111].

In the case of persistent inflammation during a wound-healing disorder, myofibroblasts can also express MCP-1 in addition to the cell types already mentioned, as could be shown using the example of hepatic myofibroblasts (LI90) [112]. MCP-1, in turn, can induce the expression of ECM-degrading matrix metalloproteinases (MMPs), such as MMP-9 [113], which is a key protease in wound healing and is normally highly expressed during the inflammatory early stages of wound healing. Moreover, this protease is also important for angiogenesis in the proliferative stage [114] and for re-epithelization in the late remodeling phase [115,116,117]. The relationship between MCP-1 and MMP-9 could be shown in a cell system comprising MCP-1-stimulated human aortic smooth muscle cells (HASMCs), where respective inhibition experiments showed that, mechanistically, MCP-1-induced MMP-9 expression requires signaling, which is based on the MAP kinases ERK1/2 and p38 [118]. Although many studies indicate that MMP-9 contributes to the formation of fibrosis in chronic wounds, information on how MMP-9 does so is sparse. In reviews on the functions of MMPs in fibrosis, the activation of TGFB1 (see myofibroblast maturation) by MMP-9 is discussed as a contributing factor [119,120]. Another issue may be that MCP-1-induced MMP-9 promotes leukocyte and particularly neutrophil transendothelial migration (extravasation) [121]. Against this background, neutrophils are a source of pro-inflammatory cytokines, like tumor necrosis factor alpha (TNF-α) [122], which support the maintenance of the inflammatory milieu, thereby favoring the establishment of fibrosis.

However, it could be shown that, in connection with fibrosis, the activity of FAK is involved in the expression of MCP-1 in fibroblasts and thus potentially in the expression of MMP-9 as well [104,113]. Further investigations proved that the inhibition of FAK in in vitro-cultured murine keratinocytes derived from FAK KO mice leads to persistent MMP-9 expression and thus to delayed wound healing in the corresponding mouse model. During delayed wound healing, MMP-9 was involved in persistent dermal ECM proteolysis, where, at the mechanistic level, Wong et al. [123] were able to demonstrate in attached and suspended keratinocyte cultures that MMP-9 was regulated by p38 MAP kinase signaling. These findings, published by the same working group of Wong et al. in 2012 and 2014 [104,123], indicate that it may depend on the wound-healing stage and, evidently, the cell type as well as to whether the inhibition of FAK is positive (MCP-1 in fibroblasts [104]) or negative (MMP-9 persistence in keratinocytes [123]) in wound healing.

Against this background, a paper published by Wang et al. [124] in 2019 seems interesting. The authors were able to show that the viability, growth, and migration capacity of fibroblasts from keloids or hypertrophic scars cultured in vitro were inhibited when the activity of FAK (FAK^p397^) was inhibited. A new finding was that FAK activity was not inhibited by pharmacological, small-molecule, or RNAi intervention but rather by apigenin, a flavonoid, found in numerous fruits and vegetables [124]. In contrast, another paper published in 2019 showed that the small-molecule-based stimulation of FAK activity (FAK^p397^) accelerated epithelial wound closure under in vivo conditions, as well as in vitro. After excluding off-target effects by the small molecule, Wang et al. [125] demonstrated that the faster wound closure observed in vivo compared to the cell behavior observed in vitro is not due to proliferation stimulation but rather due to increased Caco-2 cell migration.

#### 2.3.3. Short Summary

In the fields of wound healing, fibrosis, dermal ECM proteolysis, and wound closure, open research questions primarily concern (i) what (cell type), (ii) when (time), and ultimately also how (with what), which need to be addressed experimentally in the future in such a way that new clinically useful strategies can emerge from this. With regard to the first two points, i.e., what and when, hydrogel-based approaches, such as the spatial–temporal directed release of FAK-inhibiting small molecules [126], can be tentative options, or, with a focus on how/with what, in addition to substances that directly inhibit FAK, they can also be applied to indirectly downregulate the activity of FAK. An example of this is the inhibition of mechanoresponsive PIEZO ion channels, such as PIEZO1 since PIEZO1 co-localizes with integrin β1 in FAs, whereby FAK signaling is activated by the PIEZO1 integrin axis during mechanotransduction. By employing human proximal tubular cells (HK2 cells), Zhao et al. [127] were able to show that PIEZO1 inhibition via RNAi prevented FAK activation (FAK^p397^) and the pro-fibrotic HK2 phenotype (characterized by, e.g., TGFB1 expression, which, in turn, induced PIEZO1 expression) in the clinical context of renal fibrosis.

Taken together, the examples discussed here show that YAP/TAZ seem to play a key role in tissue regeneration during wound healing. In the skin context, for example, YAP/TAZ are involved in the maintenance of basal cell proliferation in intact as well as wounded skin, as corresponding knockdown studies have shown. Both molecules also contribute to post-wounding ECM reconstitution through their influence on TGFB1. In bone fractures, YAP/TAZ coordinate the expression of the bone-innate TF Runx-2, as well as collagen expression. In the regenerative colon epithelium, YAP/TAZ are responsible for cyclin D expression, indispensable for cell proliferation.

Regarding FAK, its inhibition can abolish MCP-1 in wound fibroblasts and thus the development of fibrosis, an aspect important during the early stages of wound healing. On the other hand, the inhibition of FAK can lead to the upregulation of MMP-9 in wound-associated keratinocytes, which leads to the overstimulation of ECM degradation, particularly dermal proteolysis in the late remodeling phase of wound healing. That, in turn, results in delayed wound healing or even the establishment of a chronic wound. Another interesting point with respect to FAK in wound healing may be that FAK activation is required for the expression of vascular endothelial growth factor (VEGF) receptor 2 (VEGFR2) [128], an important component of angiogenesis and thus neovascularization in later wound-healing stages (see also Section 2.4.2, FAK in Cancer). For this reason, it is important that the role of FAK is considered from different perspectives, i.e., above all, taking into account the respective stages of wound healing and the respective cell types, e.g., epithelial keratinocytes or connective tissue fibroblasts. This is significant, because the literature discussed here illustrates the positive and negative effects of FAK inhibition or FAK activity on wound healing. To avoid this obvious dilemma, alternative strategies, which are currently still in the experimental phase, may be a prospective option as a paradigm for MMP-9, which can be directly inhibited through hydrogel-based RNAi release [129], and this might be prospectively transferred to a FAK-specific strategy. The advantage of such a strategy is that it can be spatiotemporally directed and is, therefore, capable of addressing both aforementioned issues, the respective wound-healing stage as well as the respective cell type. Similar approaches would then also be conceivable for YAP/TAZ since both molecules are involved in the expression of TGFB1, a key molecule in the conversion of fibroblasts to myofibroblasts and, thus, a molecule that decisively contributes to the initiation and establishment of fibrosis during wound-healing disorders.

### 2.4. YAP/TAZ and FAK in Cancer from the In Vitro Point of View Using Different Cell Systems

#### 2.4.1. YAP/TAZ

Given YAP’s central function as a regulator of cell behavior, it is not surprising that changes in its state of activity have far-reaching consequences for the cells and tissues of our bodies. As described in the previous section, there is increasing evidence that YAP may also play an important role in cell and tissue aging, as it appears to be a master regulator of nuclear stability and senescence. In the context of cancer, YAP/TAZ dysregulation is associated with a variety of cancer entities in the human body. These include breast cancer, glioblastoma, hepatocellular carcinoma (HCC), mesothelioma, non-small-cell lung cancer, osteosarcoma, pancreatic ductal adenoma carcinoma, and prostate cancer, to name a few [130,131]. Various mechanisms have been described for the cause of YAP dysregulation in cancer, such as the somatic-mutation-related dysfunction of the Hippo signaling pathway, a previously described cellular YAP control instance [132]. This is exemplified by large tumor suppressor kinase 2 (LATS2) in mesothelioma [133] and LATS1/2 in pancreatic cancer [134]. Amplification of the YAP gene or its deletion is described as a further cause of YAP dysregulation. Depending on whether there is a gain (amplification) or a loss of function (deletion) of YAP, YAP obviously acts as a tumor promoter, that is, as an oncogene [135], or as a tumor suppressor [136]. Overholtzer et al. [135] identified a YAP amplicon in mouse mammary tumors and mimicked amplicon-related YAP overexpression by introducing the YAP gene into an immortalized but non-tumorigenic mammary epithelial cell line (MCF10A; following gene transfer, MCF-10A-YAP). Subsequent studies revealed that MCF10A-YAP cells displayed a proliferation advantage in response to EGF, which led to epithelial–mesenchymal transition (EMT), and were able to inhibit apoptosis compared to non-transfected controls [135]. On the other hand, Yuan et al. [137] found the loss of YAP expression in breast cancer tissue specimens and created a functional correlate to this situation by short hairpin RNA knockdown of YAP in various breast cancer cell lines, including MDA-MB-231, MCF-7, T47D, BT474, and SKBR. YAP-depleted breast cancer cell lines displayed the suppression of apoptosis in conjunction with increased migration and invasiveness. These examples make it clear that YAP dysregulation contributes to multiple facets of malignancy (discussed below), and, as will be clarified later, they are mechanistically related as well. Similar to YAP, overexpression has also been described for TAZ, as exemplified by cervical cancer. In vitro experiments on the consequences of TAZ overexpression revealed that its overexpression in HeLa cells led to a TAZ-regulated increase in programmed cell death ligand (PD-L1/CD274), which is involved in the attenuation of the host immune response to tumor cells, thus pointing to an obvious contribution of TAZ involvement in immune escape [138]. In combination, TAZ overexpression and an increase in PD-L1 yielded a reduction in apoptosis and enhanced cell proliferation and invasion, features that are among the facets of malignant tumor cells [138].

In connection with the fact that a change in the biomechanical properties of the extracellular environment entails changes in the mechanobiological properties of cells, it is an established concept in the cancer field that solid tumors show a desmoplastic reaction, including the formation of a collagen-rich connective tissue matrix [139]. In combination with the cross-linking of matrix proteins, these characteristics of desmoplasia lead to an increase in matrix rigidity. As mentioned in Section 2.1, the activity of YAP/TAZ is regulated by the rigidity of the matrix surrounding the cell. Therefore, it makes sense that the tumor-associated changes in matrix stiffness may influence the activity of YAP in such a way that YAP co-regulates multiple facets of malignant cancer cells. These include, for example, the already-mentioned proliferation and invasion, EMT promotion, cell death reduction, and multidrug resistance, as well as the induction of stem cell attributes [140].

In this context, the question arises as to how YAP/TAZ contribute to the control of tumor-associated matrix changes that cause the multifaceted nature of tumor cells just mentioned. To answer this question, digression into diseases such as pulmonary hypertension, a deadly vascular disease that is associated with increased matrix stiffness in the early stages, can be helpful. Related to this disease, in vitro studies on pulmonary arterial adventitial fibroblasts (PAAFs) have revealed that YAP/TAZ are involved in the upregulation of matrix molecules, such as fibrillar collagens, and matrix cross-linking molecules, like lysyloxidase (LOX). Betero et al. [141] were able to show that the upregulation of collagens and LOX was mechanistically dependent on the activity of a microRNA, mir-130/301, which, in turn, was controlled by YAP/TAZ activity. In addition, they were able to show that in this cell system, an increase in matrix rigidity, which was caused by collagen abundance and cross-linking, led to an increase in YAP/TAZ activity and collagen synthesis, as well as LOX expression, forming a positive reinforcement circuit [141]. The causal involvement of YAP/TAZ in tumor-associated desmoplasia is a clear indication of the mechanistic role of YAP/TAZ in the cell behavior of malignant tumor cells, which also implies their proliferation activity caused by an increase in ECM stiffness. In addition, Panciera et al. [142] found a correlation in the ECM stiffness context between oncogene-expressing cells and their surrounding extracellular matrix. Here, RTK–Ras oncogenes trigger a disproportional cellular response to the mechanical properties of the cell’s environment, such that when cells experience even subtle supra-physiological extracellular-matrix rigidity, they are converted into tumor-initiating cells. These regulations rely on YAP/TAZ mechanotransduction, and YAP/TAZ target genes account for a large fraction of the transcriptional responses downstream of oncogenic signaling [142].

Regarding the previously mentioned facets of malignant tumor cells, it could be shown using the example of YAP in colon carcinoma cell lines (SW620 and HCT116) that YAP is causally involved in EMT. RNAi experiments showed that YAP in these cell lines regulates the expression of the zinc-finger transcription factor Slug (SNAI2), which, in turn, controls the initiation of EMT by downregulating epidermal cadherin (E-cadherin) located in the AJs [143].

Using the human gastric cancer cell lines MKN 45 (which normally does not express PAR1) and MKN74, it could be demonstrated that the expression of protease-activated receptor-1 (PAR1/F2RL1) [144] in MKN45-PAR1-transfected cells or constitutively PAR1-expressing MKN74 cells leads to the inactivation of the HIPPO signaling pathway and thus to the accumulation and activation of YAP. This PAR1-induced YAP activation correlated with the increased expression of the breast cancer resistance protein ABCG2 (ATP-Binding Cassette Subfamily G Member 2) and the phosphoglycoprotein (p-GP), both of which belong to the ATP-binding cassette (ABC) transporter protein family and are involved in multidrug resistance (MDR) by regulating the ejection of chemotherapeutics from the cell [145]. Using the human hepatocellular carcinoma cell line BEL/FU, another study reported that high activity of YAP was associated with a decrease in reactive oxygen species (ROS) production [140]. The decreased ROS production caused constitutive MTOR (mechanistic target of rapamycin kinase) activity that protected cells from autophagy-induced cell death [140]. In this context, it is important to mention that ROS are synthesized intracellularly by chemotherapeutic agents and thus induce autophagy-induced cell death through apoptosis. These examples illustrate that YAP is involved in different ways in increased resistance to chemotherapy drugs (MDR). In addition, they are also an indication of and a possible explanation for how YAP counteracts cell death and thus enables malignant cancer cells to survive.

In 2014, Song et al. [146] performed experiments on different human esophageal cancer cell lines, namely, FLO-1, SKGT-4, BE3, OE33, JHESO, OACP, YES-6, and KATO-TN, and primary mouse embryonic fibroblasts (MEFs). Using these cells, they were able to show that YAP mediates attributes similar to stem cells, such as the formation of cell spheres and escape from senescence (this means prolonged proliferation capacity in vitro). Mechanistically, they elucidated that the engagement of YAP in the acquisition of these stem cell attributes in this case is based on the YAP-induced upregulation of sex-determining region Y (SRY)-box 9 protein (SOX9) expression, whereby the interaction of YAP with TEAD and the binding of TEAD to the SOX9 promoter was obligatory [146]. Of note, SOX9 is a member of the SOX family of TFs, which are developmental regulators that possess high-mobility group (HMG) box DNA-binding and transactivation domains and regulate cellular functions including lineage restriction and terminal differentiation [147].

Within the process of cancer, one of the hallmarks of malignancy is the invasion of malignant tumor cells into the neighboring tissue, that is, the ability of malignant cells to grow without respecting the organ boundary [148]. In this regard, in vitro studies using different pancreatic cancer cell lines have shown that YAP contributes to the invasiveness of cells analyzed in the Matrigel-based invasion assay. This was reported for the human pancreatic cancer cell line PANC-1, which has an inherently high expression of YAP and in which the use of YAP RNAi simultaneously reduced YAP expression and invasive capacity. Conversely, Yuan et al. [137] were able to detect a simultaneous increase in YAP expression and invasion capability in the pancreatic cancer cell line PANC-1, which only weakly expresses YAP, by introducing YAP-specific cDNA. These findings strongly suggest that high YAP expression is associated with the susceptibility to invasion and thus the malignancy of tumor cells. Another characteristic of malignant tumor cells, in addition to the obligatory criterion of invasion, is the optional criterion of metastasis [148]. To be able to metastasize, malignant cancer cells must infiltrate the endothelial blood or lymphatic system, i.e., invade one of the two vascular systems. In connection with this invasion process, Liu et al. [149] reported findings that show that YAP induces the leukocyte-specific integrin β2 in malignant cancer cells and that this leukocyte mimicry facilitates the cell invasion of tumor cells into the endothelium. The YAP-induced induction of integrin β2 in a malignant melanoma cell line with epithelial morphology (A375) enabled these cells to exhibit the transendothelial invasion of an in vitro-cultured cell layer comprising human umbilical vein ECs (HUVECs). On the mechanistic side, the authors were able to prove in their investigations that YAP, as a co-transcription activator, not only classically binds to TEAD as a TF in this case, but also interacts with a TF that has not yet been typical of YAP, namely, PR/SET domain 4 (PRDM4), in order to induce the expression of the β2 integrin [149].

#### 2.4.2. FAK

The cytoplasmic protein tyrosine FAK is overexpressed and activated in several advanced stages of solid cancers [150]. FAK promotes tumor progression and metastasis through its effects on cancer cells, as well as stromal cells of the TME. Considering the FAK-inherent features, both the kinase function within the cytoplasm and the scaffolding function inside the nucleus seemingly contribute to cancer progression [103,150,151,152] (for more details, see Section 2.5.2). With respect to the subcellular localization of FAK, cellular stress, e.g., mechanical force and oxidative stress, or the inactivation of FAK through inhibitors promotes FAK translocation to the nucleus [153]. The nuclear action of FAK requires its FERM domain, which contains nuclear export and nuclear localization signals [60]. Independent of its kinase activity, FAK required the FAK FERM F1 lobe to bind to the tumor suppressor p53, the FERM F2 lobe to achieve nuclear localization (see Figure 2), and the FERM F3 lobe to connect to MDM2, followed by MDM2-dependent p53 ubiquitination and proteasomal degradation [60]. This nuclear-FAK-driven degradation of p53 with the participation of MDM2 is an example of FAK’s scaffolding function. This example illustrates that nuclear FAK not only contributes to the loss of cell cycle control and thus unrestricted proliferation during cancer cell transformation, despite intact p53, but also promotes the survival of cancer cells by evading apoptosis [60,150,152,154]. Regarding FAK’s effects on the TME, FAK, for instance, regulates VEGFR2 and VEGF expression and, therefore, supports angiogenesis in triple-negative breast cancer (TNBC) and TNBC-derived MDA-MB-231 and MDA-MB-468 cell lines, as demonstrated by the HUVEC tube formation assay in vitro [155]. Of note, explicitly, nuclear FAK has been demonstrated to regulate VEGFR2 transcription in angiogenesis in adult mice [128,155,156]. Another, somewhat older study by Hwang-Bo et al. [157] from 2012 suggests that FAK phosphorylation and thus FAK signaling are important for the proliferation and tube formation of angiogenic cells. This could be demonstrated in in vitro studies of HUVEC and lymphatic ECs (LECs), since FAK inhibition by canstatin (a collagen-type IV-based inhibitor of angiogenesis) inhibited the proliferation and tubal formation of both cell entities. Mechanistically, canstatin inhibited angiogenesis and lymphangiogenesis via the suppression of integrin-mediated FAK signaling, with the latter being induced by pro-angiogenic angiopoietin-1 (ANGPT1). These findings can be taken as an indication that FAK plays a role in both blood vessel (angiogenesis) and lymphatic vessel angiogenesis (lymphangiogenesis). This notion was backed up by more recent findings by Hwang-Bo et al. [158], who showed that 3-O-acetyloleanolic acid (an oleanolic acid derivative isolated from the seeds of the cowpea Vigna sinensis K., known as an angiogenesis inhibitor) inhibited proliferation and tube formation in cultured human lymphatic microcapillary ECs (HLMECs) concomitant with the inhibition of FAK phosphorylation [157,158]. The central role of FAK in tumor angiogenesis has been increasingly elucidated. This is because, in a study by Pedrosa et al. [159], it could be shown that EC-inherent tyrosine phosphorylations of the FAK molecule at different positions, namely, Tyr-397 and Tyr-861 (FAK^p397^ and FAK^p861^), have different consequences on early- and end-stage tumor angiogenesis. Using FAK^p397^ and FAK^p861^ knockout mice, together with endothelial-cell-based in vitro spheroid cultures established from them, the authors were able to show that in the case of FAK^p397^ knockout, tumor angiogenesis was reduced permanently, i.e., in both the early and late stages of tumor formation. On the other hand, with FAK^p861^ knockout, disturbed angiogenesis was limited to early tumor stages, while end-stage tumors exhibited angiogenesis recovery [159].

One feature of tumor cells is their ability to escape from apoptosis. As demonstrated earlier in BT-474 and BT-20 human breast carcinoma cell lines by Kurenova et al. [160], one mechanism through which FAK enables tumor cells to evade apoptosis lies in absorbing the death receptor complex interacting protein (RIPK1). RIPK1 binds to death receptors like tumor necrosis factor receptor superfamily member 6 (FAS) on the cell membrane and is a serine–threonine kinase that contains a death domain. In their study, the authors were able to demonstrate that FAK interaction with RIPK1 inhibits staurosporine (staurosporine is a wide-range protein kinase inhibitor)-induced apoptosis by preventing RIPK1 from initiating FAK displacement from FAs, as well as FAK dephosphorylation and degradation, where FAK displacement leads to cell detachment and apoptosis [160].

Regarding the contribution of FAK to the acquisition of cancer stem cell (CSC) attributes, Fan et al. [161] were able to show that increased FAK expression caused by promotor hypomethylation led to the expression of stem cell markers in liver carcinoma cell lines. In terms of the mechanism, the authors were able to show that an abundance of β-catenin in the nucleus, which indicates β-catenin’s function as a TF rather than a mechanotransducing AJ constituent, was responsible for the acquisition of stem cell properties. Due to its correlation with CSC markers and the detected overexpression of FAK in HCC tissue, which is also associated with increased tumorigenicity, lower overall survival, and increased recurrence, the authors regard FAK as a prognostic/diagnostic marker in HCC [161].

Another characteristic of tumor cells is that they increasingly evade the growth control of the surrounding tissue. Within the framework of this progressive autonomy, proliferation also occurs in an uncontrolled manner [148,162], for example, independently of the presence of proliferation-inducing or stimulating growth factors. This circumstance can be illustrated by a study by Zhang et al. [163], in which the contribution of FAK to tumor cell proliferation in esophageal squamous epithelial carcinoma (ESCC) was examined in vitro using ESCC-derived cell lines. In the course of their investigations, Zhang et al. [163] were able to show that glucose-induced growth-factor-independent ESCC cell proliferation required the phosphorylation of FAK at histidine residue 58 (FAK^poH58^,glucose-induced) and that this phosphorylation was mediated by the activity of nucleoside diphosphate kinase 1 (NME1). At a mechanistic level, the findings revealed that FAK^poH58^ enables the binding of retinoblastoma protein 1 (RB1, in addition to p53, identified as a novel binding partner of FAK-FERM) to the FERM domain and that this scaffolding function of FAK stimulated DNA synthesis in and the proliferation of ESCC cells. Corresponding FAK^poH58^ mutations were unable to mediate the RB1–FAK FERM interaction and resulted in a failure to increase proliferation. A proof of concept of the newly discovered role of FAK in ESCC cell proliferation was provided by the increased detection of FAK^poH58^ and NME1 in corresponding tumor tissues in situ [163].

#### 2.4.3. Short Summary

Taken together, the studies discussed here illustrate that mechanobiologically relevant molecules, particularly YAP/TAZ and FAK, are key elements in the regulation of tumors, including those that are malignant and can metastasize. It is worth mentioning that this regulatory influence affects not only the tissue or cells from which the tumor arises but also the cells of the tissues surrounding the tumor, i.e., the TME. In addition to tissue specimens and animal experiments, in vitro cell-based test systems in particular have made decisive contributions to the progressive acquisition of knowledge of the molecular relationships and mechanisms of this regulation at the mechano-molecular level.

### 2.5. Future Prospects Regarding YAP/TAZ and FAK in Cancer Diagnosis and Therapy

#### 2.5.1. YAP/TAZ

Targeting YAP. Prospective clinical–therapeutic strategies with a focus on YAP intervention strategies may either address its phosphorylation status or prevent its transition to the nucleus. For instance, the photosensitizer verteporfin, used in age-related macular degeneration [164,165], could be shown to, among other things, reduce the YAP protein level and prevent nuclear translocation through the upregulation of the 14-3-3 protein, with the latter responsible for YAP binding and cytoplasmic retention, followed by proteasomal degradation [8,166]. Although very limited, a clinical study from 2014, which applied photodynamic therapy (PDT) with verteporfin, exhibited good results in terms of efficacy and safety in a phase I/II study of locally advanced pancreatic cancer through an increase in tumor cell necrosis [167,168].

YAP inhibitors. Another promising approach to inhibiting or preventing the activity of YAP could be the administration of a small molecule, in this case, the small molecule CA3, related to esophageal adenocarcinoma (EAC). In vitro assays employing, for instance, the EAC cell line SKGT-4 (which shows intrinsically high levels of YAP expression) and Flo-1, as well as an in vivo xenograft mouse model, revealed anti-tumor effects through the inhibition of YAP/TEAD-transcription-associated proliferation and an increase in tumor cell death. Under in vitro conditions, CA3, in comparison with verteporfin, showed high specificity in reducing YAP expression and transcriptional activity in SKGT-4 cells, as indicated by the downmodulation of the YAP-regulated SOX9 TF. Moreover, CA3 reduced CSC attributes in radiation-resistant EAC cells (irradiated Flo-1 cells/Flo-1 XTR) by inhibiting tumor sphere formation. Finally, CA3 suppressed EAC tumor growth in a mouse xenograft model, whereby tumor cell growth suppression was attributed to the inhibition of YAP and SOX9, which are important for CSC maintenance [169]. Moreover, Francisco et al. [170] revealed that, in the myocardial infarction (MI) context, YAP is activated in cardiac fibroblasts in response to non-reperfused MI, as well as angiotensin II (SERPINA8) stimulation. Using fibroblast-restricted genetic inactivation of endogenous YAP, the authors were able to show that YAP deletion attenuates myocardial fibrosis and cardiac dysfunction in response to MI. Mechanistically, they reported that YAP binds to the myocardin-related transcription factor A (MRTF-A) gene at putative TEA domain transcription factor (TEAD) recognition sites and induces MRTF-A expression to facilitate myofibroblast transition and profibrotic gene expression [170]. Another study is closely linked to the malignant phenotype of tumor cells and plays a role in their migration, invasion, and metastasis. Here, the researchers demonstrated that the activation of MRTF-dependent transcription correlates with FAK activation in various tumor cells, thus meaning that the elucidation of the correlation between MRTF and FAK activities would be an effective therapeutic target in the field of tumor cell migration [171].

YAP signaling modulation. A conceivable strategy to prevent the oncogenic properties of YAP could also consist of negatively modulating the signaling of G-protein-coupled receptors (GPCRs) (see PAR1, Section 2.3), which are involved in multiple cancer entities [172]. This is due to the finding that, in bladder cancer, for example, mutations in G-proteins, which bind to the GPCRs, particularly the mutation of the G-protein α_13_ (Gα_13_), are involved in bladder carcinogenesis through the activation of YAP, as explored in HEK293T and NIH3T3 cells as in vitro model cell systems [173]. However, this example already very impressively shows that it will be very difficult to develop a universal strategy for controlling YAP in the context of cancer, since the activation mechanisms of YAP are very diverse and, therefore, tissue-specific or are determined by the intrinsic properties of the corresponding tumor entity.

Targeting TAZ. An important aspect that needs to be considered is that YAP does not stand alone, but, in concert with its paralog TAZ, it also determines the behavior of cells and, as described in Section 2.3, cancer cells. This is of far-reaching importance, since it could be shown that, for example, the transcription factor SOX2, which, through interaction with TEAD4, is causally involved in the mediation of stem cell properties in tumor cells derived from head and neck squamous carcinoma, is regulated by TAZ as well [174]. YAP, on the other hand, can not only serve as a regulator of SOX2 (see Section 2.3) but also be regulated, among others, by SOX2, as shown in studies by Zhao et al. [175] on human retinoblastoma stem-like cells (Y79). Here, the binding of SOX2 to the YAP promotor, followed by its transcriptional activation, was responsible for the degree of formation of cell spheres, with the latter indicating a stem cell attribute (see Section 2.3).

For anti-cancer strategies that address YAP/TAZ activation, however, it should be noted that these two molecules also play an important role in non-cancerous situations, such as tissue homeostasis and wound healing of the skin [91], as described in Section 2.4. Furthermore, YAP is also involved in the ontogenesis of mammals, and, as explained at the beginning of Section 2.3, it may have not only tumor-promoting properties but also tumor-suppressing ones. These examples make it clear that YAP seems to play an ambivalent role in cell fate, which is determined to a considerable extent by the time axis and the spatial localization of the corresponding tissue. This shows that an intervention targeting YAP in terms of cancer prevention should be carefully considered; i.e., it should take place in a spatially and temporally controlled sequence.

As described in Section 2.3, YAP/TAZ are involved in multiple cancer entities and usually correlate with poor prognosis regarding aspects such as patient survival and tumor recurrence [130,131]. Against this background, it is obvious to analyze these two master mechanobiological molecules for their potential as targets in the context of the diagnosis, prognosis, and therapy of malignant tumors. In this context, Wang et al. [176] developed an elastic-net-based machine learning approach to build predictive models for the YAP/TAZ target score to elucidate the key Hippo pathway components and cancer drivers affecting or associated with the pathway activity in various cancer types. With their in silico approach, they found that, among the Hippo genes, the most important regulators were the mRNA expression levels of YAP1, TAZ, and TEADs. Specifically, elevated YAP/TAZ expression levels caused by somatic copy number alterations (SCNAs) were selected with a strong preference in their models of squamous-cell-involved cancer, supporting YAP/TAZ amplification as a key driver of these cancer types. Moreover, they conducted a global examination analysis of 19 Hippo core genes across 33 cancer types using multidimensional “omic” data from “The Cancer Genome Atlas”. They characterized Hippo pathway activity by a YAP/TAZ transcriptional target signature of 22 genes and, through the robustness of their data, emphasized the importance of Hippo signaling in squamous cell cancers. Based on the robust prognostic power of these 22 genes, the authors concluded that this YAP/TAZ transcriptional gene signature represents a precious tool for potential clinical application [176], e.g., the aforementioned diagnosis, prognosis, and therapy.

The possible perspective of being able to use YAP/TAZ as a diagnostic marker is supported by the recurrent HCC after transarterial chemoembolization (TACE). Here, Qian et al. [177] found that, in recurrent HCCs, approximately 83% of the patient tissues examined showed strong to very strong immunoreactivity for YAP1. TACE is a combination of chemotherapy and vaso-occlusive medication that allows the chemotherapeutic agents to remain in the blood vessels supplying the liver tumor for as long as possible. This procedure is also known as chemoembolization and involves placing a catheter in the hepatic artery [177,178].

Another tumor entity in which YAP could act as a diagnostic marker and therapy target in the future is lung adenocarcinoma (LUAD), particularly when high glucose levels are detected. For this purpose, Xue et al. [179] employed in vitro cell systems consisting of the A549 and H1 299 non-small-cell lung cancer cell lines. With the help of these cell systems, the authors were able to demonstrate, among other things, that ISGylation of YAP, i.e., covalent binding of the interferon-stimulated gene 15 (ISG15) protein [179,180], increased YAP stability and thus led to YAP overexpression, which was associated with a reduction in apoptosis. Mechanistically, they found that ISGylation at lysine residue (K) 497 is causally responsible for increasing YAP stability and thus overexpression, since the knockdown of the ISG15 protein led to the increased ubiquitination and degradation of YAP, with K497 also being one of the ubiquitination regions present within the YAP protein. Through YAP depletion experiments, the authors could further demonstrate that the glucose-metabolism-related pentose phosphate pathway (PPP), known to promote cancer, is causally triggered through YAP overexpression, since YAP in conjunction with TEAD4 stimulated the gene expression of 6-phosphogluconolactonase (PGLS), one of the decisive enzymes within the PPP. Xue et al. [179] were able to show the importance of their YAP-associated results for the clinic, i.e., possible options in diagnosis and therapy, by the fact that the degree of expression of YAP ISGylation showed a positive correlation with the detection of PGLS mRNA, especially in tissue samples of high-glucose LUAD.

#### 2.5.2. FAK

Regarding disease diagnosis and therapy, we now turn to the second key mechanobiological molecule discussed in this review article, namely, FAK. As described in Section 2.4.2, increased FAK expression in HCC, by increasing nuclear β-catenin, is implicated in poor prognosis in terms of tumorigenicity, overall survival, and recurrence [161]. Based on these properties, FAK may be used as a diagnostic marker in this tumor entity in the future. Hence, to gain an understanding of how complex the situation in the development and design of FAK-related anti-tumor strategies is, it is important to first consider the mechanisms by which FAK contributes to malignant tumor progression. Furthermore, the cellular- and tissue-associated characteristics should be considered.

*FAK as a diagnostic marker.* These points can perhaps be explained as follows. Regarding the mechanisms and the cellular- and tissue-associated characteristics, which have already been mentioned in Section 2.4.2, FAK is overexpressed and increasingly activated in many human cancers and cancer cell lines. Among the molecular mechanisms that yield the overexpression and increased activity of FAK, gene amplification, mRNA upregulation through promotor hypomethylation (e.g., HCC, [161]), and splice variants have been characterized so far, with the latter yielding over-activated FAK isoforms, as exemplified by colorectal cancer [181]. Furthermore, among the FAK-related effects on cancer progression, combinatorial FAK-kinase- and non-kinase-related effects appear important. This has been demonstrated for pancreatic ductal adenocarcinoma (PDAC) cell lines in vitro and in vivo (nude mouse xenografts), in which both the FAK kinase function and the FAK scaffolding function are required to maintain stem cell attributes in either BxPC-3, Capan-1, or MIA PaCa-2 cells [182]. Moreover, in cells in the TME, the nuclear localization of FAK has been evidenced to support a cancer-progressive microenvironment, e.g., through the downmodulation of anti-tumor immunity, thereby creating an immunosuppressive TME. This silencing of anti-tumor immunity was mediated through the control of respective chemokine gene expression, which, among others, was demonstrated in vitro in squamous cell carcinoma cells derived from mouse skin carcinoma [183].

Regulation of FAK expression. Taken together, this complexity of FAK action in cancer theoretically allows for different starting points for potential FAK-addressed anti-cancer strategies, i.e., (i) FAK expression per se, (ii) FAK translocation to the nucleus, and (iii) FAK kinase and scaffolding functions.

Since this review seeks to shed light on the importance of in vitro cell systems for the key mechanobiological players YAP and FAK, it should not go unmentioned that at the same time, with the help of these numerous in vitro cell systems, a considerable number of small-molecule-based FAK inhibitors have been identified. Most of them address the ATP-binding site within the FAK kinase domain and are now under study in preclinical or clinical phase I and phase II trials. In addition, there are also approaches that pursue FAK inhibitors in combination with either chemotherapeutics or monoclonal antibodies. The currently available FAK inhibitors, including those that address the nuclear FAK function, their combinatorial application possibilities, and their testing, as well as their tolerability and effectiveness in respective preclinical or clinical studies, can be found in recently published review articles [52,53,184,185,186,187,188] and articles specifically focused on FAK’s nuclear function [150,152]. As mentioned in Section 2.4.2, the FAK FERM domain is required for p53 degradation through nuclear FAK. Thus, FAK inhibitors that specifically address the FAK FERM domain may contribute to tumor growth prevention or inhibition, e.g., through the abrogation of nuclear-FAK-mediated degradation of the p53 tumor suppressor, which acts on p21, with the latter known as an inhibitor of cell-cycle-dependent kinases (CDKs), which are important for cell cycle progression and proliferation. In connection with the FERM domain, it is worth mentioning that recent preclinical studies, including various in vitro cell culture systems, are working on allosteric inhibitors (they generally do not bind to the ATP-binding site within the FAK kinase domain) that focus on the interaction of FAK with other proteins, such as p53 or MDM2. These allosteric inhibitors, therefore, do not address the kinase function of FAK but rather its scaffolding function (see also Section 2.4.2) [150,152]. If it were possible to establish this type of inhibitor in therapy, there would also be potent means available to have a negative effect on the tumor-promoting microenvironment via the nuclear function of FAK, since nuclear FAK, as already discussed, stimulates the expression of the VEGFR. In ECs, FAK knockdown yielded decreased EC proliferation via affecting kinase insert domain receptor (KDR/VEGFR2) expression [128]. Thus, inhibiting FAK could counteract EC-related angiogenesis, which promotes a supportive TME. This is of particular interest since it could be shown in an experimental study on lymphoma that the TME can promote tumor chemoresistance through, for instance, FAK expression in ECs in vitro and in vivo. EC-inherent FAK, in turn, protects tumor cells from DNA-damaging doxorubicin chemotherapy through FAK-triggered activation of the nuclear factor “kappa-light-chain-enhancer” of activated B-cells (NF-kB) TF and consecutive EC cytokine expression. From these results, the authors of the respective study concluded that FAK might act as a regulator of tumor chemosensitivity [189,190].

Despite their clinical application in phase I or phase II studies, most of the recent FAK inhibitors have shown only modest effects and have failed to produce objective clinical responses [191]. Against this background, the development of additional options to combat FAK in cancer progression remains very urgent. One such option could lie in targeting FAK using the so-called proteolysis-targeting chimeras (PROTACs), which render FAK harmless in cancer cells by directing it toward natural degradation via the ubiquitin–proteasome pathway. PROTACs are heterobifunctional small molecules that comprise three constituents: (i) a component binding the target protein, (ii) a unit recruiting a target-specific ubiquitin ligase, and (iii) a linker component, which connects the other two constituents. The great potential of PROTACs was illustrated by Huo et al. [192] in a recently published combinatorial in vitro/in vivo study, using ovarian carcinoma (OC) as an example. By using the OC cell lines OVCAR3 and OVCAR8 and an orthotopic OC mouse model, the authors were able to show with the help of an innovative FAK-directed PROTAC that proliferation, migration, cell survival, and invasion could be inhibited in the corresponding OC models. By selectively administering the FAK inhibitor PROTAC and its parent kinase inhibitor vs. 6063 (this is the FAK target component within the PROTAC) to OC cell cultures, the authors were able to show that the inhibitor binds to FAK and inhibits its kinase function, but the study did not address the scaffolding function of FAK. However, this was inhibited after the administration of the complete PROTAC, i.e., when the ubiquitin-ligase-recruiting unit was present. Using these cell function analyses, the authors were able to demonstrate that PROTACs are highly selective for FAK and that they target both the kinase and scaffolding functions of FAK [192]. However, there are still obstacles that stand in the way of this hopeful perspective of PROTACs regarding FAK and for cancer progression in general. These obstacles arise, inter alia, from the structure-related high molecular weight of PROTACs and their resulting unfavorable pharmacokinetics. In addition, due to the recruitment requirement for a specific ubiquitin ligase, undesirable off-target effects can also arise, which can influence cell, tissue, and organ physiology in an undesirable manner. Thus, an open research question with respect to PROTACs is how to minimize unfavorable aspects in the future to render PROTACs a powerful strategy against cancer. A promising approach lies, for instance, in the manipulation of PROTAC’s target-protein-binding component such that it exerts effects only at specific time points and locations. This would help to elevate their targeting ability and reduce potential toxicity. In this regard, light-inducible photo-caged PROTACs created by Xue et al. [193] showed the potential to improve their targeting function. Despite these hurdles, PROTACs have an advantage over traditional small-molecule-based inhibitors in that they can overcome the development of resistance in cancer cells caused by the mutation of the target protein. The concept of PROTACs, initially described by Sakamoto et al. [194], as well as the present knowledge and the future of PROTACs in cancer therapy, has been recently reviewed [195,196].

#### 2.5.3. Summary

Taken together, for all three molecules, YAP/TAZ and FAK, previous in vitro and in vivo studies, as well as studies on in situ tumor tissue, described and discussed in Section 2.4 and Section 2.5, have shown that it is very difficult per se to develop suitable therapeutic approaches. This is because all three molecules are embedded in extremely complex mechanobiological–biochemical (e.g., growth factors) signaling networks, which are also essential for physiological cell and tissue situations. Irrespective of this, small-molecule tools, with the help of suitable in vitro cell systems, seem to be a promising design platform for being able to therapeutically address all three molecules. This design platform may be successful in developing individualized treatment options in the future, i.e., tailored to the respective tumor type and possibly also to the stage of tumor progression.

The approach published by Wang et al. [176] in 2018 using omics databases to generate a transcriptional target gene signature for the signaling of certain molecules in cancer entities may, in the future, be used for both diagnosis and therapy at the same time. This is because, with the help of such an omics-based data analysis, both upstream and downstream partners of cancer-relevant signaling molecules of interest can be identified. The identification of such molecules could then lead to small-molecule-based molecular interventions with or without having to directly address the respective signaling molecule of interest, such as YAP/TAZ or FAK.

## 3. Methods

This review, written with a focus on in vitro cell systems, aims to show the reader the inestimable value and contribution of such cell systems in relation to the scientific and clinical knowledge available today and the associated achievements in mechanobiology using the examples of YAP/TAZ and FAK. To achieve this goal, an extensive and intensive study of the literature was required, for which the authors usually resorted to text-based meta-databases, such as the National Library of Medicine (NLM), with references from the entire field of biomedicine. Some of the literature cited in this review focused very precisely on individual aspects of mechanobiological relevance in the area of molecular–mechanistic functioning, whereas other aspects, such as those in the area of diagnosis and therapy, were kept somewhat more general for technical reasons. Against this background, in addition to the primary literature, reviews were also used to explain and discuss the respective aspects. Regardless of whether it was the primary literature or review articles, the literature search was based on a systematic approach, which involved the use of specific search terms for the respective overall or partial aspects. This procedure was used in all sections with a focus on (i) history, i.e., identification and characterization of YAP/TAZ and FAK, (ii) aging and senescence, (iii) wound healing, (iv) cancer, and (v) diagnosis and therapy. Due to the complexity of YAP/TAZ and FAK in terms of mechanobiology and the biomedically relevant aspects described in the sections, it was essential for reading comprehension to consult the literature that dealt with interaction partners of the target molecules YAP/TAZ and FAK.

## 4. Conclusions

The large number of in vitro cell systems discussed in this review illustrates very clearly that they have made a significant contribution to identifying and characterizing three of the key players in mechanobiology and, thus, also in mechanotransduction. This characterization includes, in particular, the functions of the molecules YAP/TAZ and FAK in the context of their own and related signaling pathways and makes it clear that these key players have an impact. This means the behavioral response, or how cells react, to mechanical–biomechanical environmental signals only in the embedded context, i.e., in a network of other signaling molecules stored upstream or downstream. This applies to the physiological situation of cells as well as to the non-physiological pathological situation, the extreme of which is the malignant tumor situation.

For all three molecules, it could be shown that they are involved in gene regulation, functioning in the cell nucleus as co-transcriptional activators, and are thus involved in the mechanobiologically induced behavioral changes in cells. With the help of corresponding investigations on in vitro cell systems, it could also be shown that not only YAP/TAZ but also FAK, in addition to gene regulation, makes a significant contribution to the integrity of the cell nucleus. Hence, issues like nuclear integrity increasingly move nuclear mechanotransduction into the focus of research. However, there are still many open research questions in this regard. This is an extremely important point, as there is initial evidence from recent studies that, for example, a senescent (SASP phenotype) tumor stroma, i.e., TME, can also influence the cytoskeletal tension and lamin A/C (LMNA)-mediated nuclear integrity of malignant tumor cells in such a way that they decrease and the tumor cells thus receive increased mechanical compliance [197].

During malignant transformation, tumorigenic transformed cells produce a changed cell environment through the manifestation of inherent genetic and/or epigenetic alterations and thus induce a change in their mechanosensitivity and mechanotransduction response, which then leads to modifications in cell behavior that ultimately determine the phenotype of a malignant tumor. In addition, following the elucidation of the molecular mechanisms with the help of cell-based functional in vitro tests, the consequences of the changed expression, activation, and subcellular localization of those three key mechanobiological players could be examined and biologically validated in more detail. This is of great importance, since the examination of corresponding tissue samples in the case of autochthonous tumors allows for correlations between the molecular status of YAP/TAZ and FAK and the respective tumor situation, but the tissue analysis does not provide any information about the biological consequences of the changes on the growth behavior of a tumor at the molecular level. In the context of diagnosis and therapy, the mechanisms of the mode of action of, for example, small-molecule-based inhibitors or PROTACs could be investigated, identified, and characterized with the help of in vitro cell cultures as far as possible and then validated preclinically.

The tissue and cell states described and discussed in this review in relation to the key mechanobiological players YAP/TAZ and FAK, namely, (i) aging and senescence, (ii) wound healing (regeneration), (iii) cancer development, and (iv) diagnosis and therapy, make it very clear that the inhibition of these molecules is not always the means of choice. This is evident, above all, from the explanations of aging/senescence and wound healing, where it could be clarified, particularly for FAK, that both inhibition and stimulation can have positive and negative effects. In the case of cancer, it must be considered that appropriate measures with regard to YAP/TAZ and FAK can send cancer cells into senescence, but senescent cells can also fuel the cancer process in an undesirable manner due to their inflammatory secretory phenotype.

## Data Availability

Not applicable.

## Supplementary Materials

The following supporting information can be downloaded at https://www.mdpi.com/article/10.3390/ijms241612677/s1.

## Abbreviations

AJs	Adherens junctions
AKI	Ischemia-related acute kidney injury
ARF	Acute renal failure
cGAS-STING	Cyclic GMP–AMP synthase–stimulator of interferon genes
EC	Endothelial cell
EM	Electron microscopy
EMT	Epithelial–mesenchymal transition
ERK 1/2	Extracellular-signal-regulated kinase
FAK	Focal adhesion kinase
FAs	Focal adhesions
FERM	Band 4.1, Ezrin, Radixin, Moesin
HFpEF	Heart failure with a preserved ejection fraction
IFs	Intermediate filaments
IRS	Interference reflection microscopy
KO	Knockout
LUAD	Lung adenocarcinoma
MCP-1	Monocyte chemoattractant chemokine-1
MDR	Multidrug resistance
miR	microRNA
MMPs	Metalloproteinases
NES	Nuclear export signal
NLS	Nuclear localization signal
NTRs	Nuclear transport receptors
p-GP	Phosphoglycoprotein
PAR1	Protease-activated receptor-1
PD-L1	Programmed cell death ligand
PDMS	Polydimethylsiloxane
PI3K	Phosphatidylinositol 3 kinase
PPP	Pentose phosphate pathway
SASP	Senescence-associated secretory phenotype
SCNAs	Somatic copy number alterations
TACE	Transarterial chemoembolization
TFs	Transcription factor
TME	Tumor microenvironment
TNBC	Triple-negative breast cancer
ZO	Zonula occludens
