# Supplementary material for: On the Value of In Vitro Cell Systems for Mechanobiology from the Perspective of Yes-Associated Protein/Transcriptional Co-Activator with a PDZ-Binding Motif and Focal Adhesion Kinase and Their Involvement in Wound Healing, Cancer, Aging, and Senescence
Source: Int J Mol Sci. 2023 Aug 11;24(16):12677. doi: 10.3390/ijms241612677 (PMC10454169; doi:10.3390/ijms241612677)
Supplement: Supplementary file 1 [file ijms-24-12677-s001.zip › ijms-2430287-supplementary.pdf]

Cell culture-based in vitro test systems for biomedical research			
**In vitro model, cell-types**	**Abbreviation or reg. name**	**Literature in vitro model**	**DOI or ATCC number**
chick embryonic fibroblasts	CEFs	Sudol, M., et al. (1995). Journal of Biological Chemistry 270(24): 14733-14741.	doi.org/10.1074/jbc.270.24.14733
immortalized mouse fibroblasts	NIH3T3	Wada, K.-I., et al. (2011). Development 138(18): 3907-3914.<br>Lee, M.-J., et al. (2014). Journal of Investigative Dermatology 134(2): 518-525.<br>Maziarz, M., et al. (2020). Journal of Biological Chemistry 295(49): 16897-16904.	doi.org/10.1242/dev.070987<br>ATCC-CRL-1658<br>doi:10.1038/jid.2013.339<br>DOI 10.1074/jbc.AC120.014698
neoplastic mouse epithelial mammary gland cells	MTD-1A	Wada, K.-I., et al. (2011). Development 138(18): 3907-3914.	doi.org/10.1242/dev.070987
human embryonic kidney cells	HEK293T	Plouffe, S. W., et al. (2016). Molecular cell 64(5): 993-1008.<br>Chang, L., et al. (2018). Nature 563(7730): 265-269.<br>Maziarz, M., et al. (2020). Journal of Biological Chemistry 295(49): 16897-16904.	doi:10.1016/j.molcel.2016.10.034<br>ATCC-CRL-3216<br>doi:10.1038/s41586-018-0658-1<br>DOI 10.1074/jbc.AC120.014698
human retinal pigment epithelial-1	RPE-1	Hu, X., et al. (2018). Oncology Letters 15(4): 4867-4872.	10.3892/ol.2018.7926<br>ATCC-CRL-4000
primary mouse aortic smooth muscle cells	MOVAS	Hu, X., et al. (2018). Oncology Letters 15(4): 4867-4872.	10.3892/ol.2018.7926<br>ATCC-CRL-2797
human embryonic kidney cells	parental HEK293	Hu, X., et al. (2018). Oncology Letters 15(4): 4867-4872.	10.3892/ol.2018.7926<br>ATCC-CRL-1573
Immortalized cervical epithelial cells	HeLa	Kim, J., et al. (2020). Proceedings of the National Academy of Sciences 117(24): 13529-13540.<br>Goñi, G. M., et al. (2014). Proceedings of the National Academy of Sciences 111(31): E3177-E3186.<br>Kanai, F.; Marignani, P.A.; Sarbassova, D.; Yagi, R.; Hall, R.A.; Donowitz, M.; Hisaminato, A.; Fujiwara, T.; Ito, Y.; Cantley, L.C. The EMBO journal 2000, 19, 6778-6791.	doi/10.1073/pnas.1917969117<br>ATCC, CCL-2<br>doi.org/10.1073/pnas.131702211<br>doi.org/10.1093/emboj/19.24.6778
epithelial-like tumorigenic hepatogenic cell line	Huh7	Kim, J., et al. (2020). Proceedings of the National Academy of Sciences 117(24): 13529-13540.	doi/10.1073/pnas.1917969117
epithelial-like tumorigenic colonogenic cell line	HT29	Kim, J., et al. (2020). Proceedings of the National Academy of	doi/10.1073/pnas.1917969117<br>ATCC, HTB-38

		Sciences 117(24): 13529-13540.	
epithelial-like tumorigenic colonogenic cell line	Caco2	Kim, J., et al. (2020). Proceedings of the National Academy of Sciences 117(24): 13529-13540.	doi/10.1073/pnas.1917969117<br><br>ATCC, HTB-37
epithelial-like tumorigenic colonogenic cell line	DLD-1	Kim, J., et al. (2020). Proceedings of the National Academy of Sciences 117(24): 13529-13540.	doi/10.1073/pnas.1917969117<br><br>ATCC, CCL-221
epithelial-like tumorigenic colonogenic cell line	SW480	Kim, J., et al. (2020). Proceedings of the National Academy of Sciences 117(24): 13529-13540.<br>Yang, J., et al. (2018). Medical Science Monitor: International Medical Journal of Experimental and Clinical Research 24: 332.	doi/10.1073/pnas.1917969117<br><br>ATCC, CCL-228<br><br>DOI: 10.12659/MSM.907782
epithelial-like tumorigenic colonogenic cell line	HCT116	Kim, J., et al. (2020). Proceedings of the National Academy of Sciences 117(24): 13529-13540.	doi/10.1073/pnas.1917969117<br><br>ATCC, CCL-247
epithelial kidney cells	LLC-PK1	Kofler, M., et al. (2018). Nature communications 9(1): 4966.	DOI: 10.1038/s41467-018-07450-0<br><br>ATCC, CL-101
mouse embryonic fibroblasts	MEFs	Deramaudt, T. B., et al. (2011). Molecular biology of the cell 22(7): 964-975.<br>Lim, S.-T., et al. (2008). Molecular cell 29(1): 9-22.<br>Ossovskaya, V., et al. (2008). FEBS letters 582(16): 2402-2406.	doi.org/10.1091/mbc.e10-08-0725<br><br>ATCC, SCRC-1040<br>doi.org/10.1016/j.molcel.2007.11.031<br><br>doi.org/10.1016/j.febslet.2008.06.004
human umbilical vein endothelial cells	HUVECs	Chen, J., et al. (2012). Kidney international 82(1): 45-52.	doi.org/10.1038/ki.2012.43<br><br>ATCC; PCS-100-010
cardiomyoblast	H9c2	Cardoso, A. C., et al. (2016). Structure 24(8): 1301-1310.	doi.org/10.1016/j.str.2016.06.003<br><br>ATCC, CRL-1446
neonatal rat ventricular myocytes	NRVMs	Nadruz Jr, W., et al. (2005). Cardiovascular research 68(1): 87-97.	doi.org/10.1016/j.cardiores.2005.05.011
human fibroblasts	WI-38	Sladitschek-Martens, H. L., et al. (2022). Nature 607(7920): 790-798.	doi.org/10.1038/s41586-022-04924-6
primary dermal fibroblasts from adult and young mice	MAFs		
primary mouse aortic smooth muscle cells	SMCs		
Human embryonic kidney cells	293T		
human dental pulp cells	HDPC	Tian, X., et al. (2022). International Endodontic Journal 55(1): 54-63.	DOI: 10.1111/iej.13636
human-derived primary skin fibroblast	HPSFs	Heckenbach, I., et al. (2022). Nature Aging 2(8): 742-755.	doi.org/10.1038/s43587-022-00263-3
diploid human foreskin fibroblasts	HDFs	Cho, K. A., et al. (2004). Journal of Biological	

		Chemistry 279(40): 42270-42278.<br>Hiroyasu, S., et al. (2017). Journal of cell science 130(14): 2329-2343.	doi.org/10.1242/jcs.196147
fetal lung fibroblasts	WI138-VA13	Nishio, K. and A. Inoue (2005). Histochemistry and cell biology 123: 263-273.	DOI 10.1007/s00418-005-0766-5
senescent human adults skin fibroblasts	TIG101		
glioblastoma cell line	U251-MG	Alza, L., et al. (2020). Cancers 12(5): 1086.	doi:10.3390/cancers12051086
glioblastoma cell line	487-MG		
non-small lung cancer cell lines	A549<br>H1299	Chuang, H.-H., et al. (2019). Frontiers in oncology 9: 22.<br><br>Xue, X., et al. (2022). Cell Death Discovery 8(1): 59.	doi: 10.3389/fonc.2019.00022<br><br>ATCC, CRM-CCL-185<br>ATCC, CRL-5803<br>doi.org/10.1038/s41420-022-00842-8
SP7/Osterix[Osx])-expressing periosteal progenitor cells	/	Kegelman, C. D., et al. (2021). Journal of Bone and Mineral Research 36(1): 143-157.	DOI: 10.1002/jbmr.4166
mouse colon epithelial cells	/	Yui, S., et al. (2018). Cell stem cell 22(1): 35-49. e37.	doi.org/10.1016/j.stem.2017.11.001
renal proximal tubular epithelial cells	RPTCs	Chen, J., et al. (2012). Kidney international 82(1): 45-52.	doi:10.1038/ki.2012.43;<br><br>ATCC, PCS-400-010
human aortic endothelial cells	HAECs	Murphy, J. M., et al. (2019). Scientific Reports 9(1): 1-14.	doi.org/10.1038/s41598-019-44098-2<br><br>ATCC, PCS-100-011
murine dermal FAK-knockdown fibroblasts	/	Wong, V. W., et al. (2012). Nature medicine 18(1): 148-152.	doi:10.1038/nm.2574.
TGFβ reporter cells	TMLC	Campbell, M. G., et al. (2020). Cell 180(3): 490-501. e416.	oi:10.1016/j.cell.2019.12.030
hepatic myofibroblasts	LI90	Dagouassat, M., et al. (2010). International Journal of Cancer 126(5): 1095-1108.	DOI: 10.1002/ijc.24800
human aortic smooth muscle cells	HASMCs	Yang, C.-Q., et al. (2014). Cellular Physiology and Biochemistry 34(2): 266-276.	DOI: 10.1159/000362997
murine keratinocytes	/	Wong, V. W., et al. (2014). Annals of surgery 260(6): 1138-1146.	DOI: 10.1097/SLA.0000000000000219
human keloid fibroblasts	HKFs	Wang, H., et al. (2019). Aging (Albany NY) 11(11): 3668.	doi: 10.18632/aging.102006
hypertrophic scars fibroblasts	HSFs		
human proximal tubular cells	HK2	Zhao, X., et al. (2022). JCI insight 7(7).	doi: 10.1172/jci.insight.152330
immortalized, but non-tumorigenic mammary epithelial cells, following YAP gene transfer	MCF-10A; MCF10A-YAP	Overholtzer, M., et al. (2006). Proceedings of the National Academy of Sciences 103(33): 12405-12410.	doi.org/10.1073/pnas.0605579103
pulmonary arterial adventitial fibroblasts	PAAFs	Bertero, T., et al. (2015). Cell reports 13(5): 1016-1032.	doi.org/10.1016/j.celrep.2015.09.049
colon carcinoma cell lines	SW620	Cheng, D., et al. (2020). International Journal of Clinical and Experimental Pathology 13(4): 701.	
	HCT116		
gastric cancer cell lines	MKN45		doi: 10.18632/oncotarget.5858

	MKN74	Fujimoto, D., et al. (2015). Oncotarget 6(33): 34788.	
human hepatocellular carcinoma cell line	BEL/FU,<br>parental BEL-7402	Zhou, Y., et al. (2019). Cancer Cell International 19: 1-15.	doi.org/10.1186/s12935-019-0898-7
human esophageal cancer cell lines	FLO-1, SKGT-4, BE3, OE33, JHESO, OACP, YES-6, and KATO-TN	Song, S., et al. (2014). Cancer research 74(15): 4170-4182.	doi.org/10.1158/0008-5472.CAN-13-3569
mouse embryonic fibroblast	MEFs		
human pancreatic cancer cell line	PANC-1	Yuan, Y., et al. (2016). Molecular medicine reports 13(1): 237-242.	DOI: 10.3892/mmr.2015.4550<br><br>ATCC, CRL-1469
malignant melanoma cell line	A375	Liu, H., et al. (2018). EMBO reports 19(6): e45180.	doi.org/10.15252/embr.201745180<br><br>ATCC, CRL-1619
triple negative breast cancer cell lines	MDA-MB-231, MDA-MB-468	Shiau, J.-P., et al. (2021). Biomedicines 9(12): 178	doi.org/10.3390/ biomedicines9121789<br>ATCC, CRM-HTB-26; ATCC, HTB-132
lymphatic endothelial cells	LEC	Hwang-Bo, J., et al. (2012). International Journal of Cancer 131(2): 298-309.	DOI: 10.1002/ijc.26353
breast carcinoma cell lines	BT-474<br>BT-20	Kurenova, E., et al. (2004). Molecular and cellular biology 24(10): 4361-4371.	DOI: 10.1128/MCB.24.10.4361–4371.2004<br>ATCC, HTB-20; ATCC, HTB-19
breast cancer cell lines	MDA-MB-231, MCF-7, T47D, BT474, SKBR-3	Yuan, M., et al. (2008). Cell Death & Differentiation 15(11): 1752-1759.	doi.org/10.1038/cdd.2008.108
esophageal squamous epithelial carcinoma-derived cell lines	ESCC	Zhang, J., et al. (2022). Oncogene: 1-12.	doi.org/10.1038/s41388-022-02568-4
esophageal adenocarcinoma cell line	SKGT-4, Flo-1	Song, S., et al. (2018). Molecular cancer therapeutics 17(2): 443-454.	doi:10.1158/1535-7163.MCT-17-0560
head and neck squamous carcinoma cell lines	HNSCC, Cal27	Li, J., et al. (2019). Cell death & disease 10(8): 603.	doi.org/10.1038/s41419-019-1838-0
human retinoblastoma stem-like cells	Y79	Zhao, N., et al. (2022). Experimental Eye Research 214: 108887.	doi.org/10.21203/rs.3.rs-836788/v1<br><br>ATCC, HTB-18
pancreatic ductal adenocarcinoma cell lines	BxPC-3, Capan-1, MIA PaCa-2	Begum, A., et al. (2017). PloS one 12(7): e0180181.	doi.org/10.1371/journal.pone.0180181<br>ATCC, CRL-1687; ATCC, HTB-79; ATCC, CRM-CRL-1420
squamous cell carcinoma cells derived from mouse skin carcinoma	SCCs	Serrels, A., et al. (2015). Cell 163(1): 160-173.	doi.org/10.1016/j.cell.2015.09.001
ovarian carcinoma cell lines	OVCAR3, OVCAR8	Huo, X., et al. (2022). Frontiers in oncology	doi: 10.3389/fonc.2022.851065<br>ATCC, HTB-161; ATCC, HTB-161
squamous cell carcinoma cells derived from uterus tissue	SiHa	Guo, Q.-s., et al. (2017). Scientific Reports 7(1): 1-12.	DOI:10.1038/s41598-017-01832-y<br><br>ATCC HTB-35
spiny mice dermal fibroblasts	/	Brewer, C. M., et al. (2021). Developmental cell 56(19): 2722-2740. e2726.	doi.org/10.1016/j.devcel.2021.09.008

**Abbreviation List**	
Abbreviation	Name
PDMS	polydimethylsiloxane
KO	knockout
FAs	focal adhesions
AJs	adherence junctions
FAK	focal adhesion kinase
IFs	intermediate filaments
ZO	zonula occludens
NTRs	nuclear transport receptors
miR	micro-RNA
NLS	nuclear localization signal
NES	nuclear export signal
EM	electron microscopy
IRS	interference reflection microscopy
FAK	focal adhesion kinase
FERM	Band 4.1, Ezrin, Radixin, Moesin
TME	tumor microenvironment
ERK 1/2	extracellular signal-regulated kinase
TFs	transcription factors
cGAS-STING	cyclic GMP-AMP synthase-stimulator of interferon genes
SASP	senescence-associated secretoma phenotype
AKI	ischemia-related acute kidney injury
ARF	acute renal failure
PI3K	phosphatidylinositol 3 kinase
MCP-1	monocyte chemoattractant chemokine-1
HFpEF	heart failure with a preserved ejection fraction
MMPs	metalloproteinases
PD-L1	programmed cell death ligand
EMT	epithelial-mesenchymal transition
PAR1	protease activated receptor-1
p-GP	phosphoglycoprotein
MDR	multidrug resistance
TNBC	triple negative breast cancer
SCNAs	somatic copy number alterations
TACE	transarterial chemoembolization
LUAD	lung adenocarcinoma
PPP	pentose phosphate pathway
